# Sedimentary Metagenomics Reveal Avian Community Transitions From the Last Glacial Maximum to the Holocene

**DOI:** 10.1002/ece3.72064

**Published:** 2026-03-27

**Authors:** M. M. Sander, K. R. Stoof‐Leichsenring, S. Liu, W. Shen, S. Lisovski, U. Herzschuh

**Affiliations:** ^1^ Polar Terrestrial Environmental Systems, Alfred Wegener Institute Helmholtz Centre for Polar and Marine Research Potsdam Germany; ^2^ Leibniz Centre for Agricultural Landscape Research (ZALF) Müncheberg Germany; ^3^ Institute of Environmental Science and Geography University of Potsdam Potsdam Germany; ^4^ Institute of Biochemistry and Biology University of Potsdam Potsdam Germany

**Keywords:** ancient environmental DNA, bird community, environmental change, glacial, palaeoecology, Pleistocene, shotgun sequencing, vegetation

## Abstract

The transition from the Last Glacial to the Holocene was marked by significant warming. This forced a compositional turnover of terrestrial plant and mammal communities discovered by diverse palaeoecological techniques. In this study, we analysed ancient environmental DNA with shotgun metagenomics from eight lake sediment cores, collected in northern Eurasia and Alaska, to elucidate the relationship of past bird communities and vegetation structure across the last 21,000 years. We leveraged all DNA reads assigned to the class ‘Aves’ to characterise the compositional changes of the bird community. The dominance of chicken birds (Galliformes, mainly ptarmigans) during the Last Glacial Maximum turned into a higher taxonomic bird diversity with increased numbers of songbird, raptor and waterfowl abundances and genera. This went along with the late glacial loss of the steppe‐tundra and the increase of shrub and tree cover. Compared to the northern boreal areas, vegetation and bird communities were more stable in the northern tundra sites, where open landscapes prevailed throughout. Metagenomics significantly contribute to the reconstruction of past avian community changes and thus have high potential to support the predictions of distribution changes in the course of future ecosystem change.

## Graphical abstract

We used sedimentary ancient DNA shotgun metagenomics to reconstruct past bird communities in northern high‐latitude environments. By using long time series and data collected across a large environmental gradient, we contributed in a unique and explorative way to the understanding of palaeoecological dynamics between birds and their habitat on the Northern Hemisphere and across different arctic biomes. We observed a shift from a dominance of open grassland birds towards a more diverse bird community during the Pleistocene–Holocene transition, along with the Holarctic vegetation composition changes.

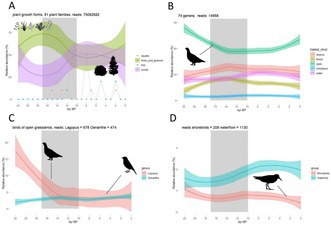

## 1 Introduction

Global biodiversity is currently experiencing one of the biggest mass extinctions in earth's history (Barnosky et al. 2011). Birds represent the most diverse group of terrestrial vertebrates, of which more than 140 bird species have become extinct in recent times and 12% are threatened with extinction (Grealy et al. 2017; BirdLife International 2019). The reconstruction of ancient ecosystems in general, and bird communities in particular, as well as the identification of the drivers of change on a large temporal and spatial scale is more important than ever to understand the mechanisms behind extinctions and community shifts to ultimately find ways to conserve biodiversity (Elias and Schreve 2016; Grealy et al. 2017; Bergman et al. 2023). Metagenomics has the potential to unravel community compositions and transitions across taxa, geographic regions and large time scales (Wang et al. 2021). This study aims to reconstruct past avian community compositions in high latitudes and elevations of the Palaearctic using sedimentary ancient DNA and assess their responses to climatic transitions.

To validate ecosystem reconstructions, it is important to understand the underlying evolutionary processes that formed the recent diversity of taxa. Birds of the Palaearctic originate from widely distributed genera with a high climate tolerance and migratory behaviour. Most of these bird species are related to open, treeless habitats. This natural selection of taxa dates back to the Pleistocene, where climate variability selected for the most resilient genera (Finlayson et al. 2012). Even though the breeding season for high‐elevation and high‐latitude birds is short and often associated with harsh and unpredictable weather conditions (Callaghan et al. 2004; Martin and Wiebe 2004; CAFF 2013), they benefit from lower competition and predation and a higher and more predictable resource availability (McKinnon et al. 2010, and references within). 313 bird species have been recorded as alpine breeding birds in Tibet, of which 67% are songbirds (Passeriformes, 210 species. e.g., *Parus*), 6% are chicken birds (Galliformes, 19 species, e.g., *Lagopus* and *Phasianus*), 7% are raptors and owls (Accipitriformes, Falconiformes, Strigiformes, 21 species, e.g., *Falco*), whilst waterfowl (Anseriformes, 15 species, e.g., *Aythya*) and shorebirds (Charadriiformes, 18 species, e.g., *Charadrius*) make up 11% of the avian biodiversity (de Zwaan et al. 2022). In northern high latitudes, about 240 bird species are breeding (Callaghan et al. 2004). Of these birds, 88 species are breeding inland and rely on terrestrial and equestrian food resources. However, in high northern latitude ecosystems, songbirds and other landbirds, such as chicken birds (e.g., *Lagopus*), raptors and owls (e.g., *Haliaethus*), play a minor role in terms of biodiversity (21%, 34 species). Overall, waterfowl and shorebirds dominate the avian diversity in the Arctic (67%, 108 species) (CAFF 2013; Smith et al. 2020). Shorebirds, e.g., sanderling *Calidris alba* , and geese, e.g., greater white‐fronted goose *Anser albifrons* , have higher abundances in the high Arctic, whereas ducks of the genus *Anas* and *Aythya* occur only up to the boreal zone (Callaghan et al. 2004). Typical for tundra and alpine grassland habitats are the migratory shorebirds *Calidris* and *Charadrius* (CAFF 2013; Smith et al. 2020; de Zwaan et al. 2022). Their worldwide decline is explained by the negative effects of climate change, which are most severe for migratory species breeding at high latitudes (Koleček et al. 2021). Less specific are raptors and songbirds. Both groups occur in diverse habitats across the gradient from open grasslands to forests. Many raptors, e.g., the peregrine falcon *Falco peregrinus* , and songbirds, e.g., northern wheatear *Oenanthe oenanthe* , are polygonally distributed from south to north (Callaghan et al. 2004). Insectivores, e.g., the northern wheatear, are almost exclusively long‐distance migrants, which is related to the availability of invertebrate food resources. An exception is the forest‐dwelling woodpecker *Dendrocopos*, syn. *Dryobates* (Morse 1971). A typical arctic raptor is the gyrfalcon *Falco rusticolus* , and within the owls, the snowy owl *Bubo scandiacus* . Both are resident and specialised on small mammals for prey (Smith et al. 2020). Chicken birds, particularly Tetraonini with the genus *Lagopus* (ptarmigan), inhabit mostly open landscapes, i.e., alpine and sub‐arctic grasslands. They are granivorous and mostly resident. These genera as a group, but the genus *Lagopus* in particular, with two species mainly confined to the Arctic and sub‐Arctic, are therefore suitable representatives of the Arctic and alpine tundra habitats and are expected to mirror changes in climate and vegetation structure (CAFF 2013; Hof and Allen 2019; Sonsthagen et al. 2022; De Amaral et al. 2023).

During the Pleistocene (2.58 million and 11,7 kyr BP), glacial periods alternated with interglacial periods, leading to species extinctions or distribution shifts towards climates and habitats in which they could endure. Both the arrival of humankind as well as environmental change are being discussed to have been the main drivers of extinction (Taberlet et al. 1998; Elias and Schreve 2016; Bergman et al. 2023). During this time, animals migrated between their glacial refugia and northern regions, and recolonisation events took place in the latter (Blondel and Mourer‐Chauviré 1998). During the Last Glacial Maximum (LGM, 26.5–19 kyr BP), the northern high latitudes were dominated by a single biome, the steppe‐tundra (dry open grasslands in permafrost regions), followed by a replacement of the herbaceous vegetation of the tundra by woody plants during the Late Glacial (19.0–11.7 kyr BP), with regional divergence of vegetation (Wang et al. 2021). For Europe, fossil records provide evidence that the avifauna of the Pleistocene was considerably characterised by different species of ptarmigans and snowy owls, which are all cold‐adapted (Mourer‐Chauviré 1993). Europe and North America experienced different changes in the distribution and extent of their biomes than Eurasia and Beringia, attributable to the divergent forming and deglaciation of ice sheets during the Pleistocene. This was leading to significantly different changes in the availability of habitats. Differences in the Late Quaternary shifts in bird community composition are likely. In Europe and North America, even the cold‐adapted species only persisted in glacial refugia, such as the Mediterranean region for Europe and Beringia for North America (Taberlet et al. 1998; Blondel and Mourer‐Chauviré 1998; Anderson et al. 2006). It is unknown whether species were present throughout the LGM due to the absence of ice sheets in Eurasia and Alaska (Beringia) (Hais et al. 2015), the regions considered in this study.

Ancient DNA extracted from sediment cores was proven to have high potential for the reconstruction of past ecosystems through metabarcoding and metagenomics, including shotgun sequencing. Particularly during the transition in the Late Quaternary, shifts in vegetation composition, as well as relationships between terrestrial and aquatic plants, plankton, fish and mammals have been quantified with these techniques, often verified with pollen records from the same cores and fossil records (Liu et al. 2021, 2024; Wang et al. 2021; Murchie et al. 2021; Bergman et al. 2023; Buchwald et al. 2024). Fossil records and ancient DNA collected in arctic regions revealed the existence of waterfowl, eagles (*Haliaethus*), falcons (*Falco*), crows (*Corvus*), cranes and allies (Gruiformes), shorebirds, tits (Paridae) and ptarmigans during the Pleistocene (Pedersen et al. 2016; Murchie et al. 2021; Courtin et al. 2022; Kjær et al. 2022). In the study of Kjær et al. (2022), a comparative approach was used in which some bird taxa were not present in any fossil records of the study site but were detected in ancient DNA. However, studies on the Holarctic bird community and how it has changed with the vast climate and vegetation transitions after the Last Glacial are lacking.

In this study, we focus on high‐elevation and high‐latitude ecosystems in Eurasia and Alaska. Before humans were reshaping these biomes during the Anthropocene, these regions were characterised by large boreal forest belts, separated by extensive steppe, desert and mountain ranges (Blondel 2022). In detail, we (1) analysed the ancient bird community compositions and their drivers in terrestrial systems of Eurasia and Alaska and (2) described the abundance change of taxonomic groups and representatives of different habitats in these regions during the Late Quaternary transition from steppe‐tundra to more diverse vegetation structures.

## 2 Methods

### 2.1 Locations and Settings of Sampled Lakes

**FIGURE 1:**
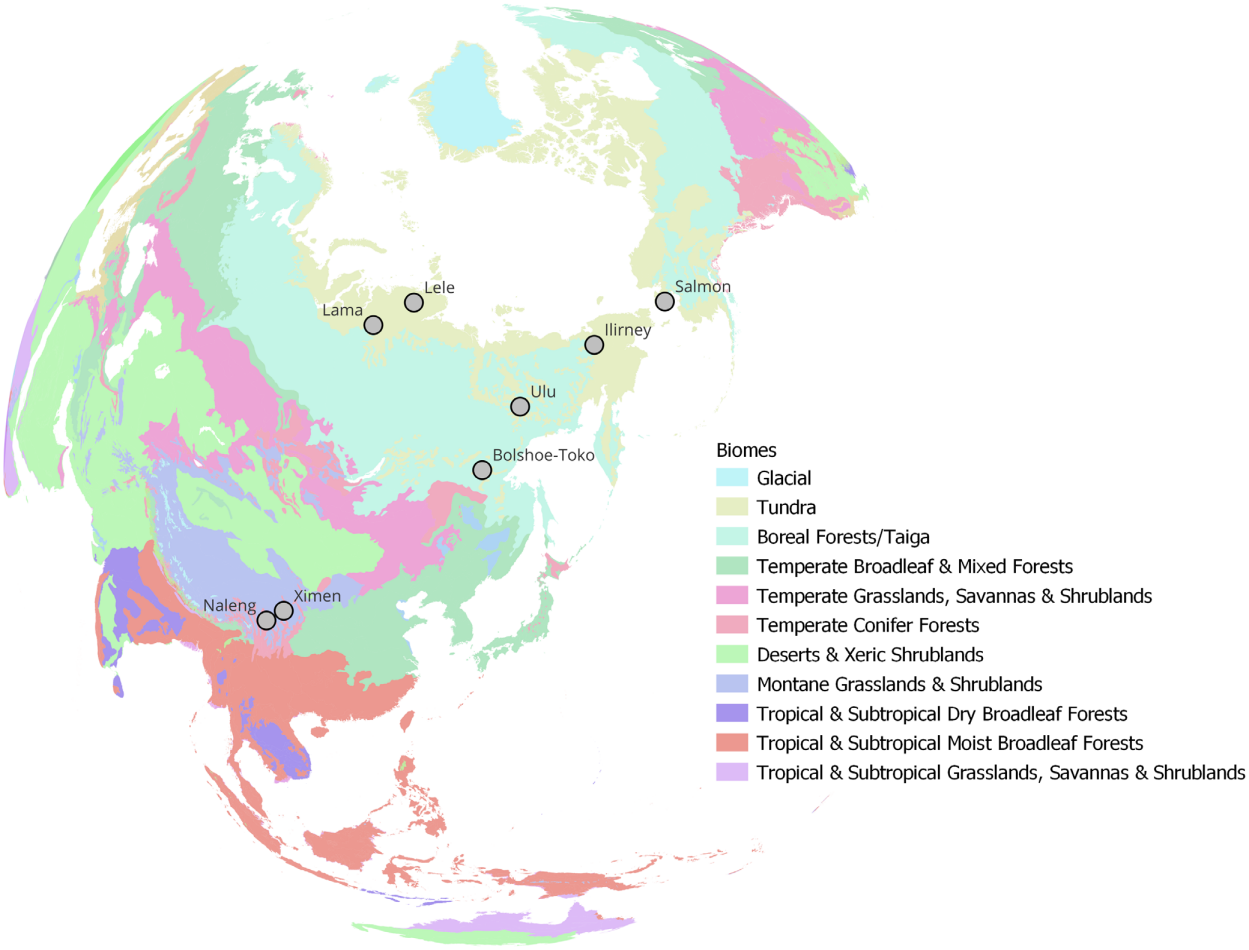
Orthographic map with the locations at which the sediment cores were extracted (eight lakes, Lele: Levinson‐Lessing). Basemaps show the biomes of the world (Dinerstein et al. 2017). Map created in QGIS version 3.34.9.

The biogeographical area covered within this study is spanning from North and Central Siberia southwards down to the Tibetan Plateau (Eurasia) and eastwards up to the Bering Strait until Alaska (Beringia), including the biomes tundra (open grasslands), taiga (boreal forest) and alpine meadows (open grasslands) (Table 1 and Figure 1). All sediment cores derive from glacial lakes. The Lakes Levinson‐Lessing (98.67° E, 74.47° N) and Lama (90.28° E, 69.55° N) are located in Taymyr, Siberia, where the climate is strongly continental (hottest month > 10°C, coldest month < 10°C). The climate is classified as polar (tundra, hottest month > 0°C < 10°C) in the former and cold summer without dry season (< 4 months above 10°C) in the latter (Peel et al. 2007). Here, the vegetation remained relatively unchanged through the Pleistocene–Holocene transition and persisted as a steppe–tundra mosaic (Wang et al. 2021). In Chukotka, Northeast Siberia (Lake Ilirney, 168.32° E, 67.35° N) and Alaska (Lake Salmon, −164.99° E, 64.91° N), with a climate classified as cold summer but with higher moisture than in Central Siberia, this transition was more prominent. During the Late Glacial, trees and shrubs became more abundant and herbaceous vegetation started to decline (Wang et al. 2021). Both lakes, Ilirney and Salmon, are located at the recent border between tundra and boreal forest (taiga). Lake Ulu in the Oymyakon region (141.05° E, 63.34° N) and Bolshoe Toko in Yakutia (130.88° E, 56.05° N) are located in Eastern Siberia, nowadays characterised by mountain taiga and a continental temperate climate with extremely cold winters and moderately warm summers. The steppe‐tundra landscape began to collapse in the Late Glacial, followed by an expansion of trees and shrubs (Biskaborn et al. 2019; Jia et al. 2024). The Lakes Ximen (101.10° E, 33.38° N) and Naleng (99.08° E, 31.17° N) are located within the Tibetan Plateau, which was characterised by alpine deserts and glacial flora after the LGM, by alpine steppe‐meadows during the Late Glacial, by mixed forests during the first half of the Holocene and alpine meadows impacted by human land use during the second half of the Holocene (Herzschuh et al. 2014; Liu et al. 2021, 2024). The geographical and climatic differences between the sites became most prominent after the LGM when the vegetation developed differently and thus provided a variety of habitats. The spatial site differences were expected to have influenced the regional occurrence and abundance of bird taxa, with, e.g., woody landscapes favouring forest birds.

**TABLE 1:** Sampling locations (eight lakes), recent vegetation (Biome), period covered by samples after subsetting for the study period from 0 to 22 kyr BP, number of samples and number of laboratory controls per core: extraction blanks (EB) and library blanks (LB).

Continent	Site (elevation)	Region (admin)	Biome	Core	Lat	Lon	kyr BP	Samples/samples 0‐22 ka/EB/LB	Age‐depth model	References of age‐depth model
Eurasia (Arctic)	Lake Levinson‐Lessing (47 m a.s.l.)	Taymyr	Tundra	Co1401	74.465	98.666	1–22	28/**15**/7/3	62	Lenz et al. (2022) (lithological, granulometric, geochemical and pollen data)
Eurasia (Arctic)	Lake Lama (53 m a.s.l.)	Taymyr	Tundra	PG1341	69.546	90.281	0–22	44/**42**/6/13	23	von Hippel et al. (2022) (fungal DNA, plant DNA metabarcoding)
Eurasia (Arctic)	Lake Ilirney (407 m a.s.l.)	Chukotka	Tundra	EN18208	67.353	168.318	0–20	17/**11**/2/3	52	Vyse et al. (2020) (sediment‐geochemical proxies, diatoms, pollen data)
Beringia (Arctic)	Lake Salmon (135 m a.s.l.)	Alaska	Tundra	EN22502	64.908	−164.989	0–21	28/**22**/9/6	30	Kaufman (2025) (age‐depth model on Pangaea)
Eurasia	Lake Ulu (950 m a.s.l.)	Oymyakon	Taiga	EN21103	63.338	141.046	3–20	23/**6**/7/4	43	Jia et al. (2024) (geochemical proxies, plant DNA metabarcoding)
Eurasia	Lake Bolshoe‐Toko (903 m a.s.l.)	Yakutia	Taiga	PG2133	56.051	130.883	0–20	27**17**/3/2	35	Courtin et al. (2021) (pollen data, plant DNA metabarcoding) von Hippel et al. (2022) (fungal DNA, plant DNA metabarcoding)
Eurasia	Lake Ximen (4000 m a.s.l.)	Tibet	Alpine grassland/shrubland	PRJEB82625	33.379	101.104	0–20	26/**26**/2/4	21	Herzschuh et al. (2014) (pollen data)
Eurasia	Lake Naleng (4200 m a.s.l.)	Tibet	Alpine grassland/shrubland	PRJEB74036	31.167	99.083	0–18	40/**40**/9/4	18	Liu et al. (2024) (shotgun data mammals, fish, plants)
	All				31–69	−164‐168	0–22			

*Note:* The age‐depth model used and references of published data of the respective lake core. Bold values are the sample sizes used in this study.

### 2.2 Core, Chronology and sedaDNA Subsampling and Extraction

The sediment core collection, dating and age‐depth model are described in previous studies (Kramer et al. 2010; Opitz et al. 2015), with further references for each lake in Table 1. Subsampling was done in a climate chamber at the German Research Centre for Geosciences (GFZ), Potsdam, Germany, under clean and cool conditions devoid of any DNA laboratory. Sterile subsampling materials, including scalpels, syringes and sample tubes, were used, and reusable sampling devices, like metallic plates and knives, were cleaned with bleach, DNAExitus, water and ethanol between the different sample depths. The final DNA samples were stored at −20°C until further use. All sample preparations for sedimentary DNA (sedaDNA) analytics were performed in the Paleogenetic laboratories at the Alfred‐Wegener‐Institute Helmholtz Centre for Polar and Marine Research in Potsdam, Germany (AWI). Clean sediment samples were stored after subsampling procedures at −20°C until the extraction of sedaDNA with the PowerMax Soil DNeasy PowerMax Soil Isolation Kit (Qiagen, GermanyMo Bio Laboratories Inc., USA) with a few modifications described in (Liu et al. 2021, 2024). Along with the sediment samples, extraction blanks (EBs) for each extraction batch (nine samples) were run. The first step of DNA isolation was slightly modified by adding 0.8 mg of proteinase K (VWR International) and 0.5 mL of 1 M dithiothreitol (VWR International) into the PowerBead tubes that were prepared with 15 mL of PowerBead solution, 1.2 mL C1 buffer and the sediment sample. Then, the solution was shaken using the FastPrep24 (MP Biomedical) for 40 s at the highest speed and then incubated at 56°C in a rocking shaker overnight. The next steps were performed according to the manufacturer's protocol.

### 2.3 Library Preparation, Shotgun Sequencing and Bioinformatic Analyses

The DNA extraction was followed by DNA concentration with the GeneJET PCR purification Kit (Thermo Fisher Scientific, Germany) and subsequent dilution to 3 ng/μL for the sediment DNA samples. In total, 30 ng of sediment DNA was used as input for the single‐stranded DNA library preparation established for ancient DNA approaches (Gansauge and Meyer 2013; Gansauge et al. 2017). Further, 3 μL of the extraction blank samples were used as input for the library preparation along with a library blank (LB), which contained solely the library preparation chemicals. A detailed full description including specific modifications to the original protocol of the library preparation can be found in (Liu et al. 2024). Final libraries were sequenced on Illumina devices using either the AWI laboratories of Uwe John at Bremerhaven, Germany or the external sequencing service Genesupport fasteris (Switzerland). Detailed information is provided in Liu et al. (2026). A compilation of the number of samples and blanks from each lake and metadata can be found in Table 1. Bioinformatic analyses were conducted as described in (Liu et al. 2024), including quality check with FastQC (version 0.7.11) (Andrews 2009), deduplication with Clumpify (version 0.20.01), adapter trimming and merging of paired‐end reads with Fastp (version 0.11.9), followed by FastQC for the merged reads. Taxonomic assignment was conducted through end‐to‐end alignment in Bowtie2, with a maximum of 1000 unique and valid alignment candidates (−k 1000) (version 2.5.1) (Langmead and Salzberg 2012). In total, our customised reference database includes 23,921,217 Aves reference genomes with 40 orders and 2321 genera. The sources of taxonomic references can be found on https://github.com/sisiliu‐research/EnviHoli. Following the HOLI pipeline (Pedersen et al. 2016), this database was constructed using Bowtie2 (version 2.5.1) with the default setting. The taxonomic classification was performed using ngsLCA (version 1.0.5) (Wang et al. 2022) with a minimal similarity of 95%.

### 2.4 SedaDNA Authentication by DNA Damage Pattern Analysis

Aves DNA damage patterns were investigated for all lakes using PyDamage version 0.72 (Borry et al. 2021), which uses the entire short read metagenomic data assembled into longer DNA fragments (contigs) by MEGAHIT version 1.2.9 (Li et al. 2015). By remapping the short reads to the contigs, PyDamage estimates the post mortem damage of the contigs, which were then taxonomically classified with Kraken2 nt 0.0. Contigs assigned to Aves were filtered for eight datasets, and the respective C to T substitution frequencies of the first 10 positions (5′ end) of those contigs that had an accuracy prediction ≥ 0.6, a contig length ≥ 1000 bp and a C to T frequency > 0 at the first position were plotted (Figure S1). For Lake Lama, the C to T substitution frequencies of the first position across sample age were investigated and are plotted in Figure S1, showing a positive relationship between the rising of C to T substitutions and increasing age. The entire dataset contained 84 blanks (sum of all extraction and library blanks), of which 67 (80%) did not build any contigs during the assembly step of the PyDamage pipeline, supporting very clean laboratory work. The remaining 17 blanks showed a very low number of contigs (median = 12), which sum to about 0.1% of the total number of contigs detected in samples and blanks. Amongst two blanks (from Salmon Lake) that had contigs, we exemplarily checked the PyDamage results. We found a very low proportion of Aves contigs (11 contigs, 0.0003% contigs from total Salmon Lake dataset). Such signals can occur from slight cross‐contamination effects or chemical contaminations during the sample preparation.

### 2.5 Taxa Selection, Data Filtering and New Rank Assignment

In a first step, all reads assigned to the class Aves (birds) in samples of the eight available lake cores were selected. From this dataset, we kept taxa that occur in at least two samples and have a total read count of ≥ 2 across all samples (of the respective lake), as in (Wang et al. 2022). With this, 50,458 reads of our targeted taxon Aves were kept across all lakes. Next, samples of all lakes aged between zero and 22 kyr BP were selected (Table 2). Controls for the DNA extraction (EB) and library (LB) were screened for Aves reads. In 58 of 84 controls (either EB or LB) Aves reads (including all reads on any taxonomic level) were detected in very low abundance (median = 4). The majority of taxa detected in the blanks belong to reads assigned to the order Galliformes.

**TABLE 2:** Number of total bird taxon reads on rank order and genus (taxa selection list Table S1).

Site	Bird order	Bird genus	*N* bird genera	Plants
Lake Bolshoe‐Toko	2312	1434	64	5,621,878
Lake Ilirney	2346	1534	63	101,857
Lake Lama	7556	4477	68	33,354,304
Lake Levinson‐Lessing	4967	2845	65	25,464,665
Lake Naleng	11,217	7914	67	2,830,130
Lake Salmon	2303	1354	64	5,530,740
Lake Ulu	474	320	55	100,938
Lake Ximen	4484	3124	67	779,491
All	35,659	23,002		Terrestrial 73,670,155 Aquatic 113,848

*Note:* Total taxon reads for terrestrial and aquatic plants on rank family (91 plant families, taxa selection list Table S1). For a number of samples, see Table 1. All taxon reads presented are aged between 22 and 0 kyr BP.

For the analyses of the bird community composition, a manual selection of all taxa identified was conducted, based on range maps from ebird.org and birdlife/IUCN (BirdLife International 2019), checking for a Palaearctic and Nearctic distribution of the species, genus or family. If reads were assigned to a species not found in these regions, it was checked whether the genus is present and, if not, whether the family is present. For example, the genus *Antrostomus* is a nightjar occurring in the New World only, but the family Caprimulgidae includes all nightjars, which do occur in the Palaearctic and Nearctic. Reads of this taxon were thus included on the family rank in all analyses. Like this, it was decided for each taxon present in the dataset whether it could be included on genus or on the family rank. In the resulting taxon selection list, the predominant habitat type (according to birdlife/IUCN (BirdLife International 2019)) was added to each taxon for later classification into groups of representatives of the major habitat types: open (e.g., *Lagopus*, *Oenanthe*), forest (e.g., *Parus*, *Ficedula*), shrubland (*Zonotrichia*), water (e.g., *Anser*, *Anas*) and diverse. The latter was used for all taxa related to more than one distinct habitat type, e.g., *Falco* (falcon) and *Hirundo* (swallow), which are found across a wide temperature, elevation, urbanisation/land use and succession gradient (see taxon selection list, Table S1). Values used for the composition analyses (principal component analysis (PCA) and distance‐based redundancy analysis (dbRDA)) are the hellinger‐transformed taxon reads (on rank genus) per time slice (rounded to kyr BP) and site ID (lake).

For the first analysis of long‐term patterns of bird communities (i, major taxonomic bird groups), all taxa assigned to Aves (using the raw dataset without taxa selection with the taxa list) were grouped on their rank ‘order’ into the four groups ‘chicken birds’ (Galliformes), ‘raptors and owls’ (Accipitriformes, Falconiformes and Strigiformes), ‘songbirds’ (Passeriformes) and ‘waterbirds’ (Anseriformes, Gaviiformes, Gruiformes, Pelecaniformes and Suliformes). Reads from all other orders were excluded from this analysis. The domestic species *Gallus gallus* (domestic chicken) and *Meleagris gallopavo* (wild turkey) showed high overrepresentation in the dataset, probably due to the high number of references in the genomic databank (Chorlton 2024, Sukhija et al. 2023) and less because of high numbers of individuals of these species in the regions under study. Nevertheless, we decided to not withdraw these reads, as the probability that the reads were still originating from the order Galliformes is high because the taxonomically nearest neighbour would be selected (Chorlton 2024). By plotting all genera of chicken birds (10 genera of Galliformes) we checked whether the domestic taxa skew the overall long‐term trend. The domestic taxa were excluded in the second analyses of long‐term patterns on genus level (see below ‘Long‐term patterns of bird communities’, ii).

### 2.6 Vegetation Structure

For each lake, all reads assigned to Streptophyta (hereafter ‘plants’) were selected and filtered with a plant taxon selection list containing all terrestrial and aquatic (macrophytes) plant families associated with arctic biomes (plant taxon selection list, Table S1). The list of plant families originates from a selection of all vascular plant species from Embryophyta clades Tracheophyta, Bryophyta, Marchantiophyta and Anthocerotophyta, which have > 10 regional occurrences in the Global Biodiversity Information Facility database (GBIF) for Siberia and Alaska (55°–90° N, 50°–150° E and 40°–90° N, 150° E–140° W) (Courtin et al. 2024). Values are relative abundances of the sum of reads of the selected families, cumulated to the growth forms ‘grasses and forbs’, ‘woody’, ‘tree’ and ‘aquatic’, for each time slice in each lake.

### 2.7 Bird Community Composition and Its Drivers

To explore spatio‐temporal bird community patterns across all sites exploratively, a PCA (function prcomp) was conducted on a subset of the above‐described data. First, the taxon selection list was used. We then filtered for reads for which the genus was known and excluded the genera *Gallus* and *Meleagris*. For each lake, this dataset was filtered for genera that occurred in at least 3 time slices of the 22 time slices, and for which a minimum of 100 reads across all eight lakes were available. This filtering resulted in a dataset of 49 genera across all lakes (Table S2.1). The matrix for the PCA was structured with the genera as columns and each row containing the reads for a single time slice for each lake. The values in the resulting matrix were normalised using the hellinger‐transformation (function *decostand* with method = ‘hellinger’). The PCA was plotted with the function *ggplot*. To identify the drivers of the bird community composition, a dbRDA (function *dbrda*, package vegan, with distance = ‘euclidean’) was conducted on the same matrix (response). Euclidean distance was chosen as variances were normalised prior to analyses, and the lakes have all genera in common (Bakker 2024). The explanatory variables were kyr BP (numeric), latitude (numeric), relative abundances of plant growth forms at time × at site × (numeric) and site (lake, factor). Multicollinearity was checked with function *cor* (package stats) and variables with correlation coefficients > 0.6 were removed. Reduced models were run and compared according to the proportion explained by the explanatory variables. Permutation tests were performed to test for the significance of the full model (function *anova*, with 999 permutations) as well as for the single explanatory variables (function *anova*, with by = ‘margin’, permu = 200).

### 2.8 Long‐Term Patterns of Bird Communities

Relative abundances are calculated as the abundances of (i) reads of the major taxonomic bird groups relative to the total number of reads assigned to Aves and grouped into the four major groups (see above) and (ii) reads of genera relative to the total number of reads in the selected dataset. An overview of relative abundances of major taxonomic bird groups (calculated as described in i) across time is presented with a stratigraphic plot, function *geom_line_exaggerate* in package tidypaleo (version 0.1.3). To show the temporal pattern of vegetation and bird community, relative abundances (calculated as described in ii) are displayed with unconstrained local regressions, plotted with the function *geom_smooth* (function = ‘loess’) in the package ggplot for (a) plant growth forms, (b) habitat groups, (c) representative genera of open grasslands (*Lagopus* and *Oenanthe*) and (d) genera of waterfowl (Anseriformes) and shorebirds (*Charadrius*, *Calidris*). For (c), we modelled the relationship of the number of taxon reads (response) and the vegetation structure with linear models over all lakes and years (function *lm*). Due to the correlation of vegetation covers with each other and with kyr BP (function *cor*, method = ‘spearman’), we reduced the model and used the percentage of grasses and forbs as the only explanatory variable.

## 3 Results

The complete dataset for all eight lakes consists of 35,659 reads assigned to the class Aves for which the order was given and which were aged between 22 and 0 kyr BP. The taxon selection list included 94 taxa with 82 bird genera (Table S1). With this list, 23,002 reads (64%) on rank genus were kept, from which domestic chicken and turkey were removed, resulting in 14,946 reads (42% of all Aves). Of the total 73,784,003 reads assigned to plants (91 plant families), only 0.15% were aquatic (Table 2).

### 3.1 Bird Community Composition and Its Drivers

**FIGURE 2:**
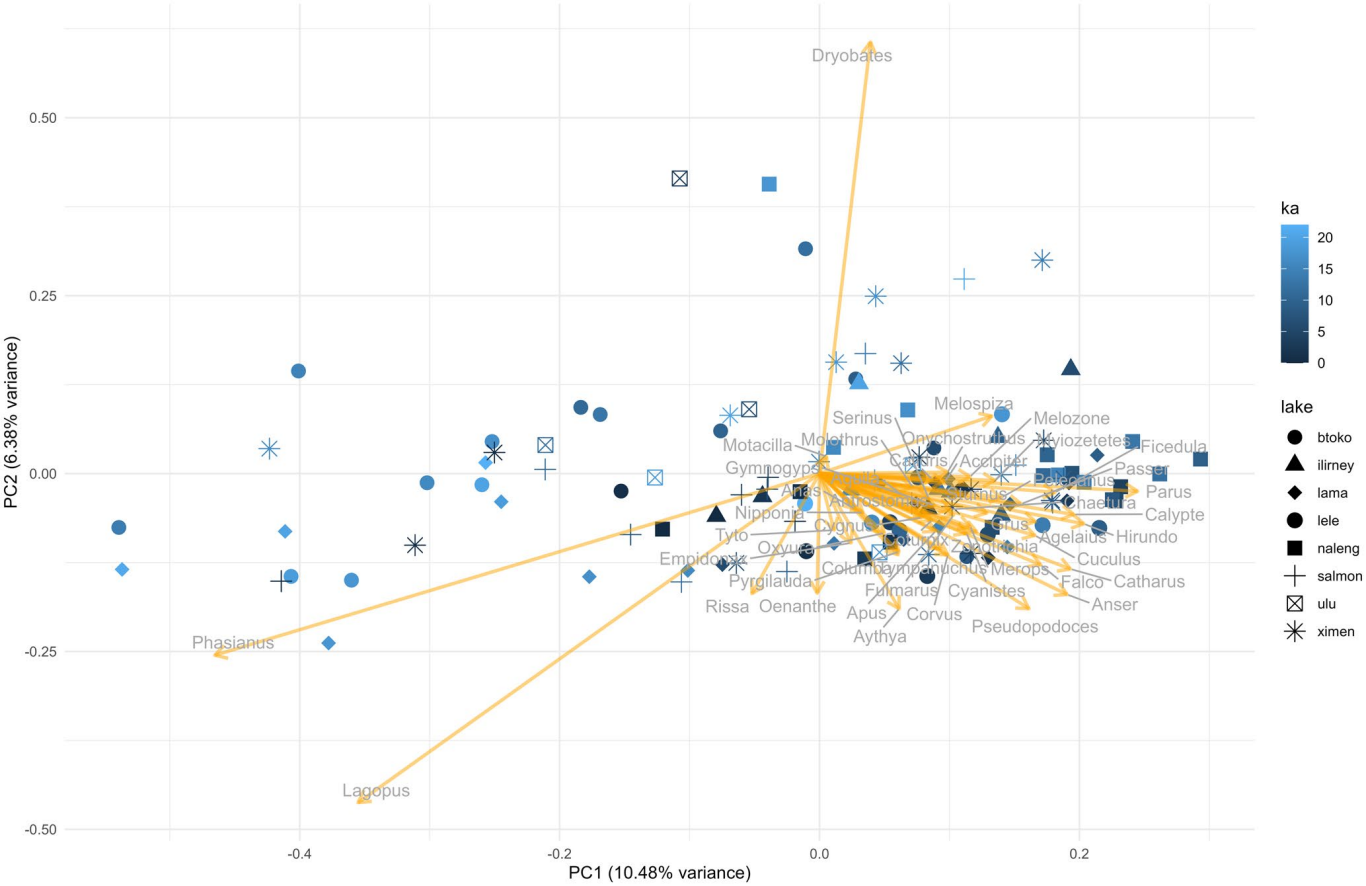
PCA of taxon reads (hellinger‐transformed) of 49 most abundant bird genera (present in at least in three time slices, minimum of 100 reads across all lakes) from eight lake sediment cores identified by sedaDNA read assignment by shotgun sequencing. The colour scale represents kyr BP (ka) and the shape indicates the lake. Details are given in Table S2.

The results of the PCA show age patterns in the occurrence of genera across the period of 22 kyr. Samples from the glacial are negatively correlated with PC1. PC1 is therefore related with time as a proxy for climate conditions. The majority of the genera (most of them are songbirds) is found in the Holocene and at southern locations (lake Naleng), whilst chicken birds (*Phasianus* and *Lagopus*) are more prominent in samples aged before the LGM and in northern locations (lakes Salmon, Lama), shown by a strong negative relationship with PC1. The woodpecker *Dryobates* is positively (Lake Naleng, Bolshoe‐Toko), whilst the wheatear *Oenanthe* is negatively associated with PC2 (Lake Levinson‐Lessing). Regarding the habitat associations of these taxa and the vegetation around the lakes, this might indicate vegetation structure (Figure 2 and Table S2.2). The dbRDA shows that variance in the taxon read abundance of genera is significantly explained by time slice, grasses and forbs (kyr BP, positive dbRDA1) and sampling locations (lakes) (Table 3). The lakes are combining various environmental factors, most importantly latitude and vegetation, and thus are important determinants of bird community structure (Figure S2). The plant growth forms ‘grasses and forbs’ and ‘woody’ were highly correlated. Thus, we excluded the variable ‘woody’ from the models. Lakes are representing the latitudinal gradient. Models with ‘lake’ were explaining more variance than the ones with ‘latitude’. We thus decided to include ‘lake’ instead of ‘latitude’. Reduced models without plant growth forms were explaining less variance (Table S3) and thus underpinned the importance of these variables to understand the structure of the bird community. Grasses and forbs are positively related to dbRDA1 and dbRDA2, whilst the trees and aquatic plants are negatively associated with dbRDA1. In general, the results of the dbRDA show that the relative abundances of genera reflect a gradient in time (dbRDA1, strong association with time slice) and space (differences between lakes), determined by the abundance of different plant growth forms as a proxy of the available habitat. Open landscapes (grasses and forbs) are related to glacial and late glacial samples, whereas trees and aquatic plants are found in the Holocene and in southern samples.

**TABLE 3:** dbRDA biplot scores for constraining variables in the global model for the hellinger‐transformed taxon reads of 49 bird genera (response). Variance explained by constraining variables: 17% (proportion) and 6% (inertia).

Component	dbRDA1	dbRDA2
Eigenvalue (accumulated)	1.77	1.04
Proportion explained	0.28	0.16
Cumulative proportion	0.28	0.44

Constraining variables	dbRDA1	dbRDA2	Sum of Sq	*F*	*p*
kyr BP	0.42	−0.07	0.47	1.60	0.003**
Grasses and forbs	0.22	0.28	0.50	1.70	0.009**
Trees	−0.23	0.11	0.3250	1.10	0.286
Aquatic plants	−0.42	0.38	0.4418	1.50	0.073.
Lake Bolshoe‐Toko					
Lake Ilirney	−0.03	0.10	4.20	2.03	0.001***
Lake Lama	0.17	−0.56			
Lake Lessing‐Levingston	−0.20	−0.28			
Lake Naleng	−0.53	0.15			
Lake Salmon	0.17	−0.26			
Lake Ulu	0.12	0.22			

*Note:* ANOVA (function *anova*, with 999 permutations) of global model: *p*‐value < 0.01. Further test statistics of the permutation tests of the global model and reduced models are given in Table S3. Test statistics (*F*, *p*‐value) for constraining variables were obtained with *anova* (dbRDA, by = ‘margin’, permu = 999). Levels of significance: * < 0.1, ** < 0.01, *** ≤ 0.001.

### 3.2 Long‐Term Patterns of Bird Communities

**FIGURE 3:**
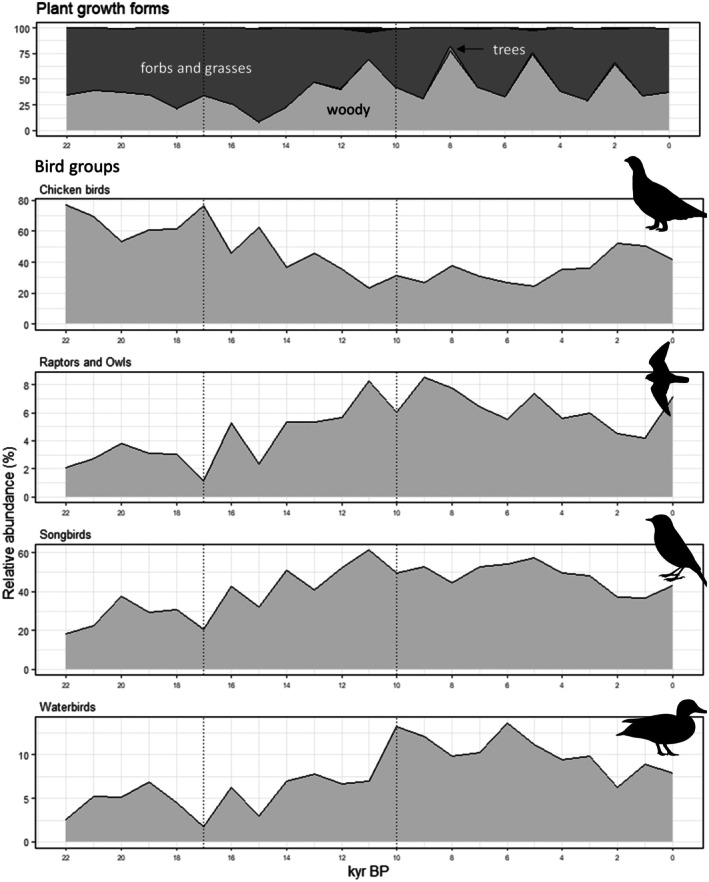
Major shifts in relative sedaDNA read assignment by shotgun sequencing through time for eight northern high‐latitude and high‐elevation lake cores. Percentages of plant growth forms as indicators for vegetation on rank ‘family’ (91 families), and major taxonomic bird groups on rank ‘order’ (11 orders). Chicken birds: Galliformes. Raptors and owls: Accipitriformes, Falconiformes and Strigiformes. Songbirds: Passeriformes. Waterbirds: Anseriformes, Gaviiformes, Gruiformes, Pelecaniformes and Suliformes. Relative abundance: taxon reads per year as % of all bird (Aves) taxon reads. Vertical lines represent the period in which the major transition of habitat and bird community was observed (17–10 kyr BP). Sample sizes are given in Table 2. Pictograms under creative common licence from phylopic.ord.

**FIGURE 4:**
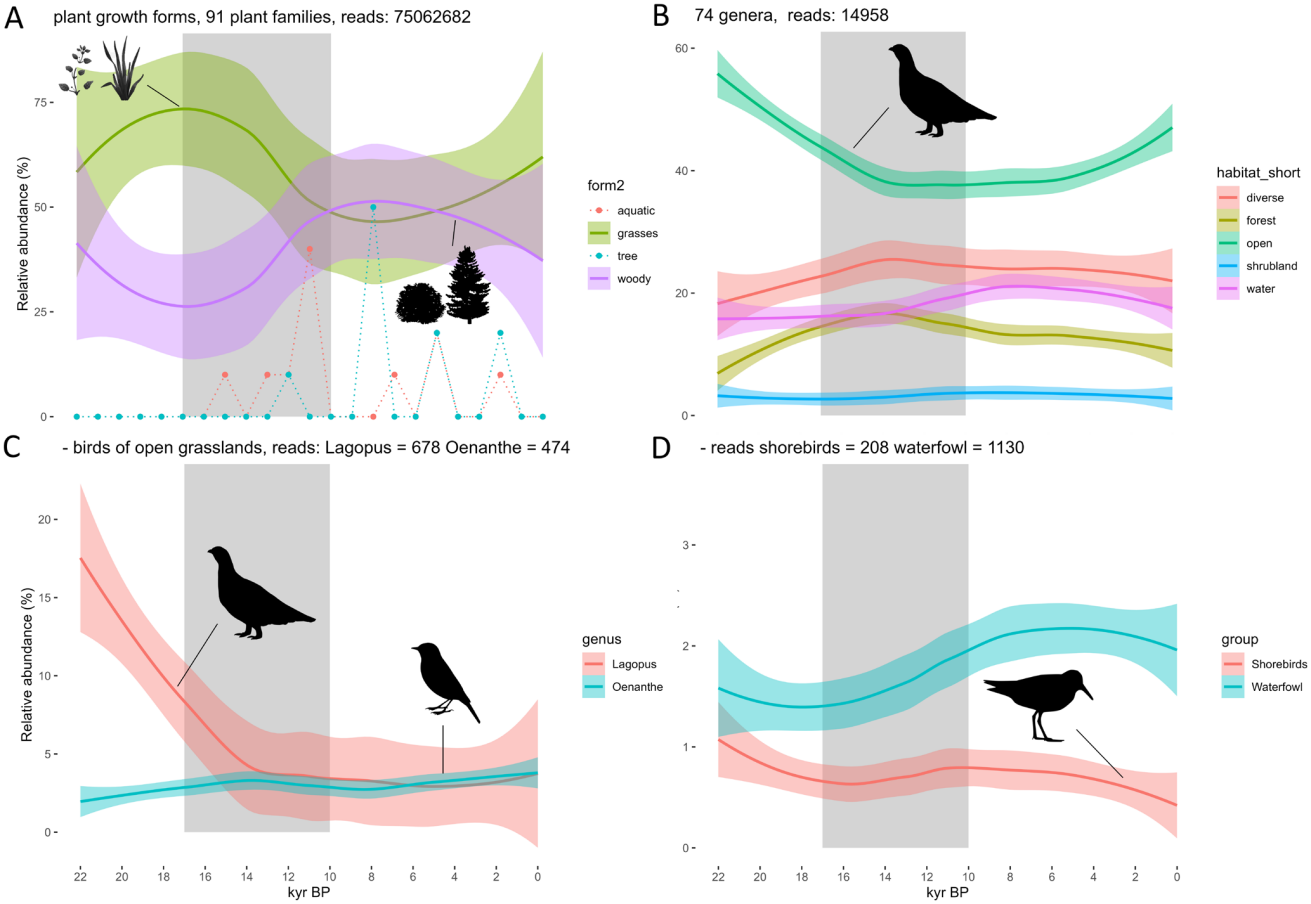
Shifts in (A) plant growth forms on rank ‘family’. Relative abundance: Taxon reads per year as % of all plant taxon reads. Forms ‘tree’ and ‘aquatic’: Rel. abundance *10. (B) Birds assigned to habitat types on rank ‘genus’ (74 genera). (C) Abundances of *Lagopus* and *Oenanthe* as representatives of open grassland habitats. (D) Abundances of shorebirds (*Calidris, Charadrius*) and waterfowl (Anseriformes) included on rank ‘genus’. Relative abundance: taxon reads per year as % of all bird (Aves) taxon reads after selection with the taxon selection list (Table S1). Local regressions plotted with *loess* function of package ggplot. Shading marks the period in which the major transition of habitat and bird community was observed (17–10 kyr BP).

At the transition from the Glacial to the Holocene between 17 and 10 kyr BP, decreases in the abundances of chicken birds (Galliformes) were observed, whilst raptors and owls, songbirds and waterbirds increased (Figure 3). Here, we kept all reads assigned to Galliformes, as the domestic taxon *Gallus* was in line with the general decreasing trend of chicken birds and showed similar temporal patterns as the ptarmigans *Lagopus* (Figure S3). The single genera of waterfowl and raptors and owls show slightly different patterns from 10 kyr BP onwards (Figure S4). We observed a transition from a forbs and grasses dominated landscape towards higher abundances of woody plants accompanied by the establishment of trees. This is mirrored by a shift from birds of open habitats towards birds of diverse and water habitats, forest and shrublands (Figure 4A,B). However, we found regional differences in these general vegetation shifts. In northern Taymyr (site Levinson‐Lessing), grasses and forbs dominate the habitats until recent millennia. In Chukotka (site Ilirney) and Alaska (site Salmon), no notable change was observed over the 21,000 years, with grasses and forbs as well as woody plants shaping the habitats around the sites. Thus, in the Arctic, tundra persisted even after the LGM. Conversely, in Taymyr (site Lama), Oymyakon (site Ulu) and Yakutia (Bolshoe Toko) and the Tibetan Plateau (sites Ximen and Naleng, as reported in Liu et al. 2021), a shift from the grasses and forbs dominated landscape towards woody plants was observed between 17 and 10 kyr BP, and the biome changed from tundra to present‐day taiga and a mosaic of alpine open and wooded vegetation. The sample size of site Ulu is much lower than that of the others. For this reason, vegetation turnover is shown as a linear relationship only but mirrors the general increase of woody and decrease of grassy vegetation (Figure S5). The two representatives of open grasslands showed opposing trends, with *Lagopus* decreasing drastically and *Oenanthe* remaining stable or increasing (Figure 4C). We formally tested these trends with linear models using the data from all lakes, showing a significant positive relationship of *Lagopus* with higher percentages of grasses and forbs (β = 0.09, df = 101, *p* = 0.005), whereas *Oenanthe* did not show a significant relationship (β = 0.01, df = 109, *p* = 0.37). In the Arctic sites Salmon and Ilirney, these changes were weaker, whilst Lake Lama and Levinson‐Lessing showed strong decreases of birds associated with open landscapes, similar to the more southern Lakes surrounded by taiga (Bolshoe‐Toko) and Ximen in the alpine habitat. The numbers of taxon reads for *Lagopus* and *Oenanthe* were not sufficient for a local regression for Lake Ulu (see plots for single lakes Figure S6). During the transition after the LGM, waterfowl and shorebirds were both increasing. This changed after 10 kyr BP, when waterfowl showed increases, whilst shorebirds were decreasing (Figure 4D, and for single lakes, see Figure S7).

## 4 Discussion

We found major differences in trends between bird groups as well as the representatives of tundra habitats. Whilst previous studies proved the existence of single bird taxa in specific epochs (e.g., Murchie et al. 2021; Kjær et al. 2022), we provided new insights on how the entire bird community changed significantly within 7000 years along with the overall ecosystem change during the transition from Pleistocene to the warmer Holocene. The bird community of the Pleistocene was characterised by birds of open habitats, most likely with a significant number of ptarmigans, and changed into a more diverse community mirroring the vegetation turnover.

Analysing post mortem damage with a few and very short DNA reads requires the accumulation of reads to longer contigs as established in PyDamage (Borry et al. 2021). The analysis of post mortem damage patterns on Aves DNA contigs revealed the expected increase of C to T changes at the 5′ end of the DNA contigs. This typical pattern confirms that the detected Aves DNA is of ancient DNA origin. Further, the C to T rate of the first position is significantly correlated with the age of the samples, supporting the assumption that older Aves DNA has higher post mortem damage patterns compared to younger samples (Figure S1). With this study, we contributed significantly to the detection of cryptic bird community shifts in relation to biome transitions by using ancient sedimentary DNA.

### 4.1 Changes in the Bird Community Composition

During the global transition to a warmer climate, the bird community became more diverse in terms of taxonomic and habitat groups. Conversely, pollen and sedaDNA metabarcoding analyses of lake sediments from Bolshoe‐Toko revealed that the steppe‐tundra supported a higher terrestrial plant species richness than the forested habitats after the transition, therefore describing a decrease in biodiversity in terms of species richness (Courtin et al. 2021). Similarly, at lake Ilirney, the highest plant richness within our period of study was found between 13 and 9 kyr BP and decreased steadily since then along with the increase in woody vegetation and the loss of the steppe‐tundra (Huang et al. 2021). However, we explain the increasing diversity of the bird community with the overall diversification of vegetation structure during the Holocene (Wang et al. 2021), assuming that plant species richness is of lower importance for birds than the structural diversity determining niche space (LaRue et al. 2023, and specifically shown for forest birds by Kebrle et al. 2023).

The strongest change in vegetation was observed from 17 to 10 kyr BP, including the cooler period of the Younger Dryas. Whilst in the steppe‐tundra of the Pleistocene, chicken birds (i.e., *Lagopus*) dominated the bird community, the habitats of the Holocene were inhabited by higher abundances of songbirds (e.g., *Parus*, *Cyanistes* and *Motacilla*), raptors and owls (e.g., *Falco* and *Tyto*) and waterbirds (e.g., *Anas* and *Anser*). The increase in songbirds was facilitated by the increasing shrub encroachment and establishment of trees. These are particularly important for the occurrence of woodpeckers *Dryobates*, which are strongly connected to shrubland or forest and are characteristic of recent boreal forests worldwide (Blondel 2022). When looking at the habitat groups, the bird community shifted from representatives of the open habitat and tundra (ptarmigans and shorebirds) towards birds of more diverse (*Falco* and *Hirundo*), water (e.g., *Anser* and *Anas*) and forest habitats (e.g., *Dryobates, Ficedula* and *Catharus*), promoted by an increase in temperature and woody and tree plant families.

### 4.2 Changes in Open Grassland Bird Abundances

The abundance changes in open grassland birds after the LGM differed between wheatears, ptarmigans and shorebirds. Depending on the magnitude of the regional shrub and tree encroachment, we furthermore found regional differences. Wheatears were common across the Pleistocene Northern Hemisphere and did not decline with climate warming during the Holocene. This genus breeds across a wide temperature gradient and thus is expected to have a high potential to adapt to climate change. For example, the Holarctic species *O. oenanthe* tolerates temperature increases at the nest sites and can adjust breeding phenology (Low et al. 2019; Jähnig et al. 2020; Dunn et al. 2020; Sander et al. 2021), but is sensitive to vegetation changes, i.e., the loss of open habitat (Alba et al. 2023). In regions where the forbs and grasses decreased after the LGM and the increase in shrubs and trees was particularly strong (e.g., Lake Ximen, Bolshoe‐Toko and Lama), we observed decreases after the LGM. In the Arctic, which were less affected by vegetation changes (Lakes Salmon, Levingston‐Lessing), trends were slightly positive after the LGM. Recent population trends of wheatears vary regionally, but in general, lowland populations are stable whilst mountain populations are decreasing due to climate warming and the related shift from alpine open grasslands towards shrubland and forest (Scridel et al. 2018; Lehikoinen et al. 2018; López‐Ramírez et al. 2024).

The second targeted open grassland taxon, the ptarmigans, was present in high numbers throughout the Pleistocene. As they are feeding primarily on grass seeds and have their recent optima in the southern tundra subzone (Callaghan et al. 2004), they have been most likely benefitting from the extensive areas of open grassland habitats across the Northern Hemisphere. The occurrence of ptarmigans at this time was also reported by Murchie et al. (2021) with aDNA and by Mourer‐Chauviré (1993) with fossil records. However, detailed information on abundance changes, as well as their abundance relative to other bird groups, was only revealed by our study. Contrary to the patterns observed in wheatears, ptarmigans, which are cold‐adapted and sensitive to temperature increases (referring to recent population decreases), began to decrease rapidly at the beginning of the Holocene at around 11 kyr BP. This negative trend was weaker in the Arctic. Recent populations of *Lagopus* are decreasing rapidly with climate warming and the decrease of cold habitats (Smith et al. 2013), particularly in the uplands and mountains, where shrub encroachment and rising temperatures lead to a drastic shrinkage of the extent of suitable habitats (Scridel et al. 2018, 2021; Lehikoinen et al. 2018).

Our observations for both genera, *Lagopus* and *Oenanthe*, imply that high‐latitude habitats provided refuge for open grassland birds during the Pleistocene–Holocene transition. Similar to the ptarmigans, we observed decreases in shorebirds (i.e., waders) at the beginning of the Holocene. They might have suffered from range contractions due to shrub encroachment during the transition, an increased predation risk for ground‐nesting species due to higher predator abundances (here raptors) and the overall warmer climate in the Holocene. These threats became even stronger towards present day, as recent population decreases are related to direct and indirect anthropogenic environmental change effects, including degradation of breeding habitats (i.e., loss of intertidal mudflats), increased predation risk, phenological mismatch, as well as multiple threats encountered during migration, such as degradation of suitable stopover and wintering habitats and hunting pressure (Meltofte et al. 2007; Smith et al. 2020; Lameris et al. 2021; Koleček et al. 2021). Our results need to be treated with caution as we had low read numbers and records of only two genera (*Calidris, Charadrius*). Furthermore, we could not differentiate trends regionally. This bird order has many species at northern high latitudes, and we expected a stronger representation of this group (only 1.2% assigned to Charadriiformes). However, their low contribution to the pool of bird taxon reads might be related to their relatively short and seasonal presence on their arctic breeding grounds (see ‘Limitations’ below).

### 4.3 Changes in Birds of Prey Abundances

Raptors and owls contributed 3.2% and 1%, respectively, to the overall number of taxon reads assigned to birds. Considering their territoriality and their relatively low abundances with less than one couple per square km (Vazhov 2015), these low contributions were expected. Cold‐adapted hawks (*Accipiter*) and bald eagles (*Haliaeetus*) did not show major abundance changes at the beginning of the Holocene. A study using lake cores sampled in Beringia also detected bald eagles in environmental DNA (throughout the Holocene) (Pedersen et al. 2016). Interestingly, golden eagles (*Aquila*), falcons (*Falco*) and barn‐owls (*Tyto*) seem to have profited from climate change (warming) in that period. Most species of the genus *Falco* are nowadays related to warmer climates and lower latitudes, despite tundra (gyrfalcon, Booms et al. 2020) and taiga breeders (merlin *F. columbarius*, Warkentin et al. 2020). Barn‐owls showed the lowest abundance when woody plants and trees were peaking, which might be related to their preference for semi‐open landscapes. Nowadays, this genus has many species in the tropics. Only the Western *T. alba* and American barn‐owls *T. furcata* are breeding in temperate zones. No species occurs in the taiga or tundra (Winkler et al. 2020a), which brings up the question of why the genus was relatively abundant in the past and if this signal is true. Potentially, the read assignment should be more general at the order level (owls). This would include the more northerly distributed genus *Bubo (snowy owls)*, which contributed significantly to the Pleistocene avifauna, as fossil records revealed (Mourer‐Chauviré 1993). Whilst falcons and owls are dependent on small mammals for prey, bigger raptors (golden eagles, bald eagles and hawks) are more generalistic in their prey but prefer bigger items, such as waterfowl, chicken birds, other birds of prey, medium and bigger sized mammals and mammal carcasses (Nadjafzadeh et al. 2016; de Gabriel Hernando et al. 2024). Increasing abundances of small mammals, ungulates and wild boars and thus increasing food resources for birds of prey were described before (Pedersen et al. 2016; Liu et al. 2021; Murchie et al. 2021), implying improved conditions for predators after the LGM. Recent threats to Arctic avian predator populations, e.g., snowy owls, peregrine falcons and gyrfalcons, which build their nests in soil cliffs of the thawing permafrost and partly winter on the decreasing sea ice, were related to climate change (CAFF 2013).

### 4.4 Changes in Waterfowl Abundances

Waterfowl abundances increased during the Holocene. This is likely related to the increase in temperature, which favoured aquatic plants and surrounding wetland plants. These were found to be most abundant in lakes and times with higher temperatures (Stoof‐Leichsenring et al. 2022) and provide food and nesting habitat for waterbirds. The genera *Anser*, *Cygnus*, *Anas* and *Aythya (Anatidae)* are relatively abundant in our data and were also found in Pleistocene Greenland (Kjær et al. 2022). They are well represented in the recent Palaearctic avifauna. However, some taxa are missing which are abundant across the Northern Hemisphere, e.g., the shovelers *Spatula* (Winkler et al. 2020b). Interestingly, our data shows a strong decrease in geese at around 6 kyr BP, which falls together with the beginning of the increasing human impact on the global environment (Olofsson and Hickler 2008; Kathleen Lyons et al. 2016; Ellis et al. 2021). This might imply a human impact on bird abundances through increased hunting pressure and habitat degradation at this time. This would be an interesting starting point for further examinations but cannot be covered in the present explorative study. Recent populations of waterfowl are increasing in arctic regions, partly because they benefit from global warming induced higher food resources for longer time periods in the arctic summer (Smith et al. 2020). Generally, we expected a higher abundance of waterfowl in our data due to their major contribution to the avian biodiversity in arctic ecosystems, but the number of taxon reads assigned to Anseriformes is relatively low (5.0% of all bird taxon reads) compared to the other bird groups (e.g., Galliformes 37.8% and Passeriformes 35.0%). The non‐detection of waterfowl and fish in ancient DNA deriving from lake cores in Beringia was reported in Pedersen et al. (2016) who used complementary methods (fossils) which then revealed the existence of these animals. Contrary to this, a study analysing ancient DNA from lake cores from Greenland found waterfowl (here Anatidae) to be the only birds found (Kjær et al. 2022). Similar to the animal taxa, the number of reads for aquatic macrophytes is much lower than for each of the three terrestrial plant groups (grasses and forbs, woody, tree). This is in line with a similar study that used ancient DNA to investigate compositional changes in macrophytes during the Pleistocene–Holocene transition. Also, here aquatic organisms were lower in numbers than the terrestrial ones (Stoof‐Leichsenring et al. 2022). Degradation of DNA might differ between autochthonous (in the lake) and allochthonous (in the catchment) sources of DNA that flow into the lake sediment (Jia et al. 2022). Thus, the interpretation of the varying patterns for waterfowl abundances in different lakes remains vague and needs further examination, eventually with additional methods to reconstruct animal occurrence, such as fossil records.

### 4.5 Limitations of Bird Sedimentary Ancient DNA Records

Although this study merged data of sediment cores from eight different lakes, the number of reads assigned to the class ‘Aves’ was relatively low (50,458 reads) compared to the number of reads assigned to other organism groups (e.g., Viridiplantae and mammals). This finding is consistent with previous studies, such as those using a single core from Lake Naleng (Tibetan Plateau, Liu et al. 2024) and permafrost sediments from the Batagay megaslump (Yana Uplands, northern Yakutia, Russia, Courtin et al. 2022). Whilst shotgun sequencing yields a relatively low number of reads for specific taxa, other molecular approaches, such as hybridisation capture with specific molecular probes (e.g., Schulte et al. 2021; Murchie et al. 2021) and metabarcoding using polymerase enzymes (Taberlet et al. 2018), have demonstrated the power of sedimentary ancient DNA to retrieve detailed genetic information, even in complex environmental samples.

In our first approach, we used all reads assigned to ‘Aves’ and grouped them into the four major bird groups on the order level. This approach minimised information loss by avoiding excessive interpretation about the presence of lower taxonomic levels. In the second approach, we based the selection on extant species, genera and families. This limits the reconstruction of the past by what is present now but allows for more detailed reconstructions on the genus level. To partly counterbalance the limitation deriving from using recent species distributions, we selected all taxa with distributions on a large geographical scale (Northern Hemisphere).

The lakes did not differ in their genus (and species) pool, although taxa occurred in somewhat different quantities. We therefore did not analyse diversity metrics, such as the Shannon index or the species richness. This limits the interpretation in a migration context and the description of patterns of recolonisation and extinction events. These can be described only roughly on the scale of biomes, not regionally.

When using sequence similarity as a rule for taxonomic classification, the representation of a (study or domestic) taxon in the reference database (here HOLI), e.g., *Gallus* (domestic chicken, 1,532,270 unique reference sequences), is a bias and influences the probability of a DNA sequence to be assigned to exactly this taxon instead of to a (closely‐related) less represented taxon (e.g., *Lagopus*, 183,668 unique reference sequences). For the analyses on genus level, we thus excluded *Gallus* and *Meleagris*. We did not exclude the genus *Phasianus*, although introduced as a game bird in the 16th century and nowadays distributed across the Palaearctic, because *Phasianus colchicus* originates from East Asia (original distribution), associated with woodlands (Draycott et al. 2008). Closely related to this are less well represented chicken birds (*Lagopus*, Squires et al. 2023) which occur at higher latitudes and might have been more abundant within our study regions than *Phasianus*. In this case, we cannot be sure that the high numbers of *Phasianus* taxon reads are related to a high abundance in the bird community or rather misassigned reads from closely related taxa (see for taxonomic structure of Phasianidae Shen et al. 2014). However, this bias may become less of a limitation in the future as bioinformatics and artificial intelligence tools are advancing (Mock et al. 2022). In the case of *Lagopus*, substantial progress in sequencing and building up reference genomes was made (Squires et al. 2023). This strengthens our finding that this group was declining drastically.

A general limitation in ecosystem reconstructions is that we cannot make assumptions of whether taxa were not present in the habitats in the lakes vicinity or not present/preserved in the sediment of the lakes. Some genera of widespread songbirds, e.g., *Phylloscopus* and *Sylvia*, have not been detected at all. The genus of the *Sylvia* warblers is a group of small insectivorous birds (with 6000 unique reference genomes in HOLI). Their occurrence was dated back to more than six million years ago. Most of the extant 17 species occur in the Mediterranean basin, known as glacial refugia for plants and animals during glacial periods (Blondel et al. 1996; Taberlet et al. 1998; Blondel and Mourer‐Chauviré 1998). Some species of this genus, e.g., lesser whitethroat *Sylvia curruca* , are well‐represented breeding birds of the Palaearctic with populations in high‐latitude and alpine habitats (Doswald et al. 2009; BirdLife International 2024). Their existence during the Pleistocene and early Holocene is therefore likely. However, we do not have empirical data about to which magnitude the body mass of the species (for songbirds rather low) and the number of individuals in the surrounding of the lake, where the sediment core was collected, are contributing to the probability to be detected by shotgun sequencing. If this was the case, why are songbirds in general more frequently detected than the larger‐bodied geese? Future studies should focus on the quantitative contributions of the number and size of organisms to the pool of reads in sedaDNA datasets to strengthen the conclusions we draw from relative taxon abundances. First conclusions are made when looking at the higher biomass of plants compared to mammals and their abundance in read count data (Liu et al. 2024). To overcome this issue, we here focussed on the relative change of abundances within and across bird groups.

Another explanation for the varying amount of taxon reads in birds is their different migratory behaviour and the amount of time present in the high latitude breeding grounds. Whereas chicken birds remain at their breeding grounds most of the time of the year (except some avoidance behaviour during winter by performing altitudinal migration), waterfowl and shorebirds, e.g., the Holarctic distributed greater white‐fronted geese, are strictly migratory, spending less than half of the year in their Arctic breeding grounds and the rest on migration and at lower latitude wintering grounds (O'Connel et al. 2006). Thus, they might have escaped detection because of lower presence time and the overall higher mortality outside of their breeding range, i.e., during migration.

### 4.6 Potential of Ancient Environmental DNA Analyses for Reconstructing Changes in Bird Communities

Whilst previous studies included single bird taxa within their studies on regional past ecosystems, we captured all available bird taxa from across large areas of the Northern Hemisphere. Despite the limits and uncertainties of ancient DNA analyses, we thus discovered major palaeoecological dynamics between vegetation and bird communities during the transition from the Pleistocene, with highly variable climate and land cover, to the Holocene with a warmer and stable climate. Our results imply that during the Pleistocene–Holocene transition from open to woody and forested landscapes, the arctic tundra provided a refuge for cold‐adapted grassland birds, of which many were declining in most regions of the Northern Hemisphere. Future studies involving ancient DNA will benefit from advances in modern DNA analyses and DNA repositories. Studies may focus on target groups, such as migratory birds or birds dependent on wetlands (waterbirds, e.g., Gruiformes), which are both threatened by recent climate change (Kirby et al. 2008; Amano et al. 2020). Furthermore, more detailed biodiversity and food‐web analyses could identify the drivers of change in ancient bird communities. Species distribution models are predicting the northward and upward range shift of northern high‐latitude and high‐elevation species and entire Palaearctic genera due to climate warming (Parmesan 2006; Doswald et al. 2009; Chamberlain et al. 2016). Ancient DNA analyses and thus the investigation of past large‐scale and long‐term changes in bird communities in response to environmental change contribute to a better understanding of how they will behave in a warming climate and ultimately changing biomes in the future.

## Author Contributions

**M. M. Sander:** conceptualization (equal), formal analysis (lead), visualization (lead), writing – original draft (lead), writing – review and editing (equal). **K. R. Stoof‐Leichsenring:** formal analysis (equal), investigation (lead), writing – original draft (supporting), writing – review and editing (equal). **S. Liu:** formal analysis (equal), investigation (supporting), writing – original draft (supporting), writing – review and editing (equal).**W. Shen:** investigation (equal). **S. Lisovski:** conceptualization (equal), writing – original draft (supporting), writing – review and editing (equal). **U. Herzschuh:** conceptualization (equal), formal analysis (supporting), investigation (lead), project administration (lead), visualization (supporting), writing – original draft (supporting), writing – review and editing (equal).

## Conflicts of Interest

The authors declare no conflicts of interest.

## Supporting information


**Data S1:** ece372064‐sup‐0001‐FigureS1‐S7‐TableS1‐S3.docx.

## Data Availability

This study uses sedaDNA shotgun datasets previously published (Liu et al. 2026). The respective raw shotgun datasets from four lake sediment cores are available under the BioProject accessions PRJEB94536 (Lake Levinson‐Lessing), PRJEB80635 (Lake Ilirney), PRJEB80642 (Lake Bolshoe Toko), PRJEB82635 (Lake Ulu) and PRJEB82717 (Lake Salmon) at the European Nucleotide Archive (ENA). Additional sedaDNA shotgun data sets from Lake Lama (PRJEB80877) (von Hippel et al. 2025) and from Lake Naleng (PRJEB74036) (Liu et al. 2024) were also included in this study. The raw data sedaDNA shotgun from Lake Ximen has been made publicly available with this manuscript and has been archived under the BioProject accession PRJEB82625. The documentation of the bioinformatic workflows, metadata, taxonomic reference indexing resources of the taxonomic database and an example script for filtering the final datasets is publicly available (Liu et al. 2026). The analysis code is available on zenodo https://doi.org/10.5281/zenodo.18836674.
