# Supplementary material for: Sedimentary Metagenomics Reveal Avian Community Transitions From the Last Glacial Maximum to the Holocene
Source: Ecol Evol. 2026 Mar 27;16(4):e72064. doi: 10.1002/ece3.72064 (PMC13107265; doi:10.1002/ece3.72064)
Supplement: Supplementary file 1 — Data S1: ece372064‐sup‐0001‐FigureS1‐S7‐TableS1‐S3.docx. [file ECE3-16-e72064-s001.docx]

**Supplementary material**

**
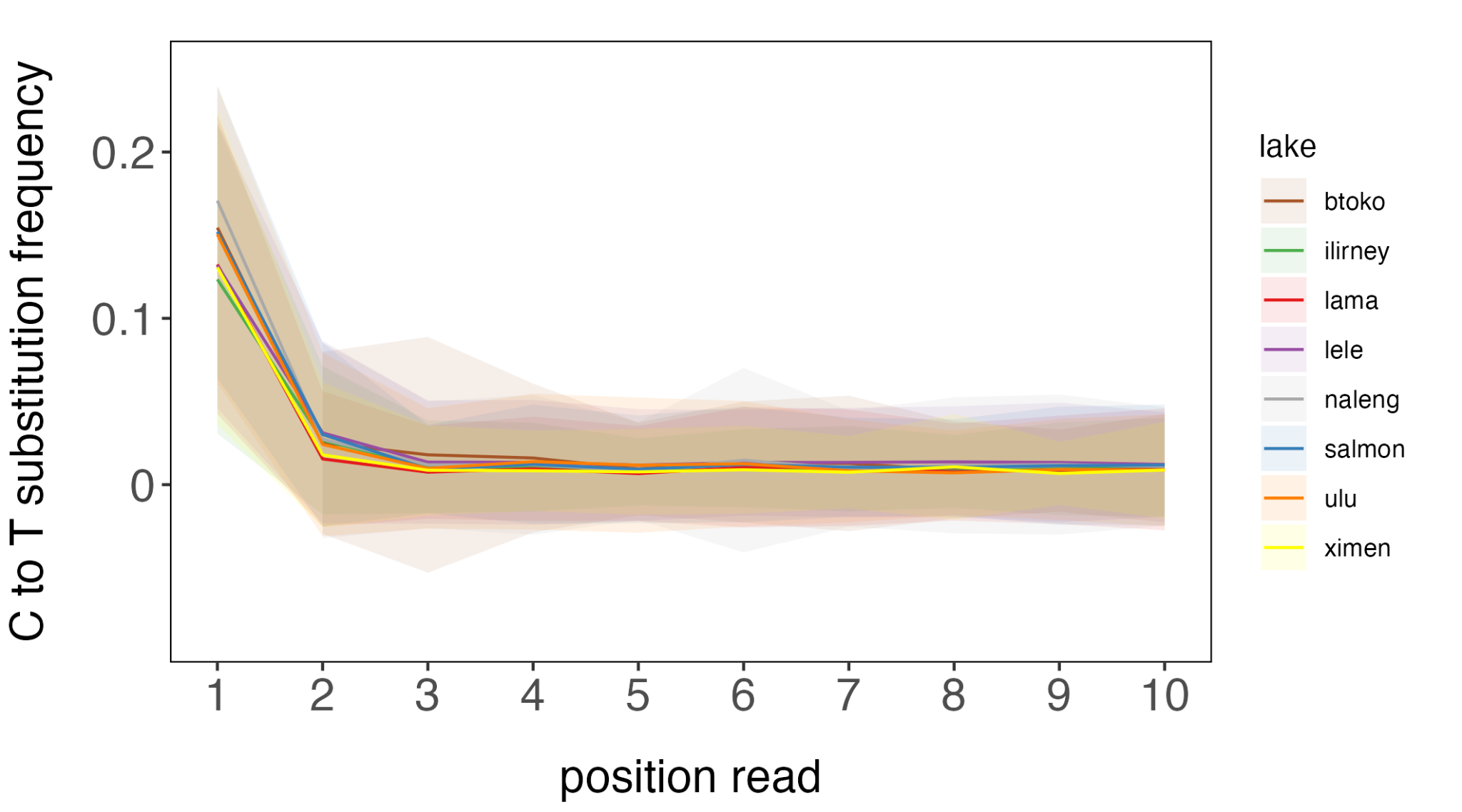
**

**
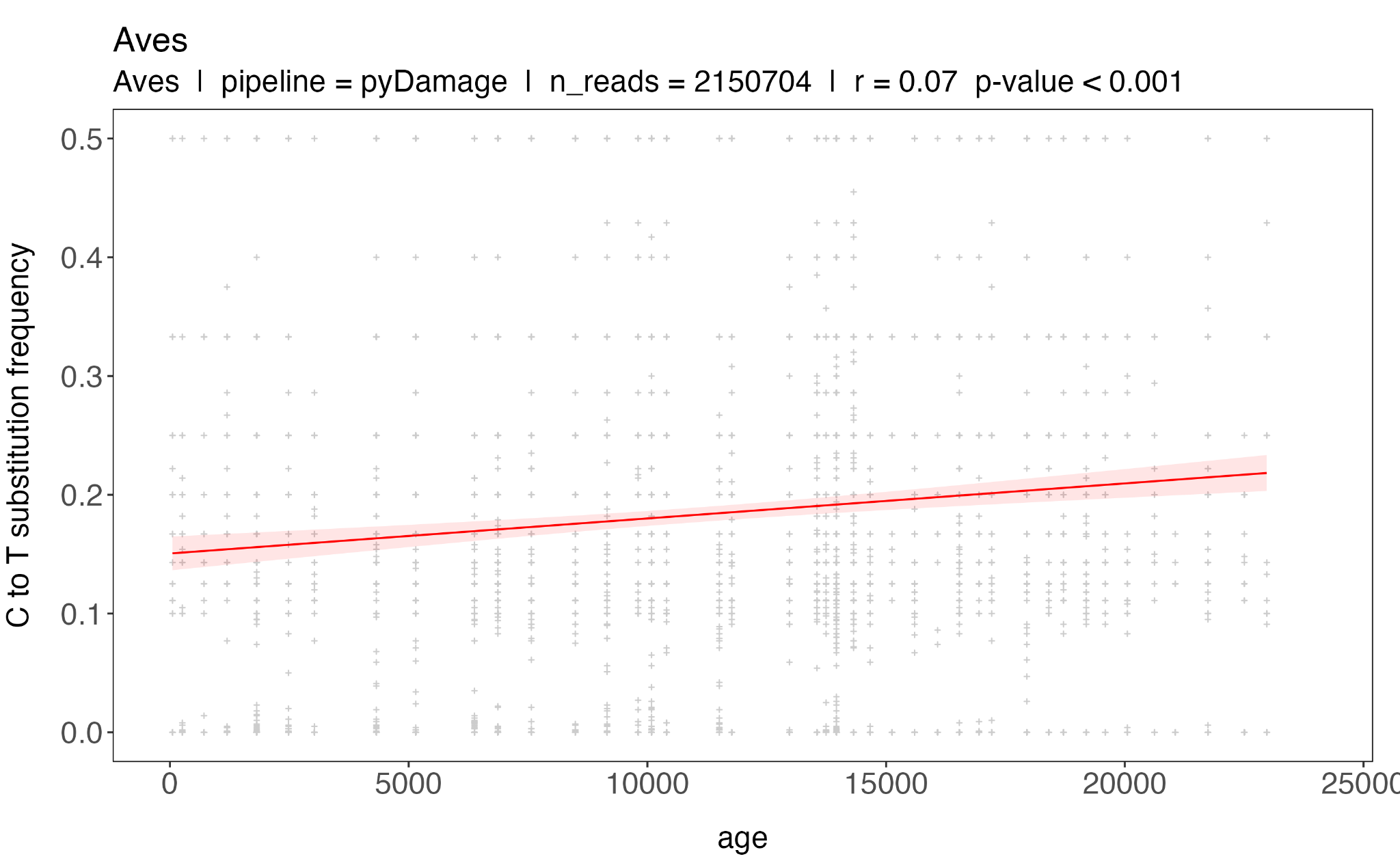
**

**Figure S1** Post mortem DNA Damage patterns in AVES contigs detected with PyDamage. **A** The frequency of C to T changes of pre-filtered (contig length ≥1000bp, prediction accuracy ≥0.6) reads for the eight lakes. **B** Relationship between C to T frequency of the first position across age exemplified on Lake Lama.

**Table S1.1 Taxon selection list** with details about distribution (general and extant (genus) in the wider study region according to descriptions in the Arctic Biodiversity Assessment^1^ and distribution maps in eBird (ebird.org) and BirdLife/IUCN^2^), habitat, migratory status, diet, likelihood to have occurred in study region covered by lake cores based on recent distribution and habitat, assignment to major taxonomic group and rank on which the taxon was included in the analyses.1.Arctic Biodiversity Assessment: status and trends in Arctic biodiversity. in *Chapter 4 Birds* (eds. Barry, T., Berteaux, D. & Bültmann, H.) (The Conservation of Arctic Flora and Fauna, Akureyri, Iceland, 2013). 2.The IUCN Red List of Threatened Species. *IUCN Red List of Threatened Species* <https://www.iucnredlist.org/en>.

| Order | Family | Genus | Distribution | Extant | Habitat | Migratory status | Diet | Likelihood | Group | New rank |
| --- | --- | --- | --- | --- | --- | --- | --- | --- | --- | --- |
| Accipitriformes | Accipitridae | Harpiia | Neotropic | no | forest | resident | carnivore | low | raptors | family |
| Accipitriformes | Accipitridae | Accipiter | Holarctic | yes | diverse | resident | carnivore | high | raptors | genus |
| Accipitriformes | Accipitridae | Aquila | Holarctic | yes | open | resident | carnivore | high | raptors | genus |
| Accipitriformes | Accipitridae | Buteo | Holarctic | yes | open | partial | carnivore | high | raptors | genus |
| Accipitriformes | Accipitridae | Haliaeetus | Holarctic | yes | water | partial | piscivore | high | raptors | genus |
| Accipitriformes | Cathartidae | Cathartes | Nearctic | no | diverse | migratory | carnivore | low | raptors | family |
| Accipitriformes | Cathartidae | Gymnogyps | Nearctic | no | open | resident | carnivore | low | raptors | family |
| Falconiformes | Falconidae | Falco | Palaearctic | yes | open | resident (Gyrfalcon) | carnivore | high | raptors | genus |
| Strigiformes | Strigidae | Athene | Holarctic | yes | open | resident | carnivore, insectivore | low | owls | genus |
| Strigiformes | Tytonidae | Tyto | Cosmopolitan | yes | open | resident | carnivore | high | owls | genus |
| Caprimulgiformes | Caprimulgidae | Antrostomus | Nearctic | yes | diverse | long-distance | invertebrates | medium | nightjars | family |
| Anseriformes | Anatidae | Anas | Palaearctic | yes | water | resident | herbivore | high | waterfowl | genus |
| Anseriformes | Anatidae | Anser | Palaearctic | yes | water | short-distance | herbivore | high | waterfowl | genus |
| Anseriformes | Anatidae | Aythya | Palaearctic | yes | water | partial | omnivore | high | waterfowl | genus |
| Anseriformes | Anatidae | Cygnus | Palaearctic | yes | water | partial | herbivore | high | waterfowl | genus |
| Anseriformes | Anatidae | Oxyura | Holarctic | yes | water | short-distance | herbivore | low | waterfowl | genus |
| Charadriiformes | Charadriidae | Charadrius | Holarctic | yes | open | long-distance | invertebrates | medium | shorebirds | genus |
| Charadriiformes | Scolopacidae | Calidris | Palaearctic | yes | open | long-distance | invertebrates | high | shorebirds | genus |
| Charadriiformes | Laridae | Chroicocephalus | Palaearctic | yes | water | migratory | omnivore | high | seabirds | genus |
| Charadriiformes | Laridae | Gelochelidon | Palaearctic | yes | water | long-distance | invertebrates | high | seabirds | genus |
| Charadriiformes | Laridae | Rissa | Palaearctic | yes | water | resident | piscivore | high | seabirds | genus |
| Procellariiformes | Procellariidae | Fulmarus | Holarctic | yes | water | migratory | piscivore | high | seabirds | genus |
| Gaviiformes | Gaviidae | Gavia | Palaearctic | yes | water | short-distance | piscivore | high | waterfowl | genus |
| Gruiformes | Gruidae | Balearica | African | no | open | resident | omnivore | low | cranes | family |
| Gruiformes | Gruidae | Grus | Holarctic | yes | open | full migrant | omnivore | high | cranes | genus |
| Gruiformes | Otididae | Chlamydotis | Palaearctic | yes | open | partial | omnivore | high | bustards | genus |
| Pelecaniformes | Ardeidae | Butorides | Holtropic | no | water | resident | piscivore | low | herons | genus |
| Pelecaniformes | Ardeidae | Ardea | Palaearctic | yes | water | partial | piscivore | high | herons | genus |
| Pelecaniformes | Ardeidae | Bubulcus | Cosmopolitan | yes | water | partial | omnivore | medium | herons | genus |
| Pelecaniformes | Ardeidae | Egretta | Palaearctic | yes | water | partial | herbivore | high | herons | genus |
| Pelecaniformes | Pelecanidae | Pelecanus | Palaearctic | yes | water | short-distance | piscivore | high | pelicans | genus |
| Pelecaniformes | Threskiornithidae | Nipponia | Palaearctic | yes | water | resident | piscivore | high | ibises | genus |
| Pelecaniformes | Threskiornithidae | Platalea | Palaetropic | yes | water | resident | invertebrates | medium | spoonbills | genus |
| Suliformes | Phalacrocoracidae | Phalacrocorax | Palaearctic | yes | water | resident | piscivore | high | cormorants | genus |
| Apodiformes | Apodidae | Chaetura | Nearctic | no | diverse | long-distance | invertebrates | low | landbirds | family |
| Apodiformes | Apodidae | Apus | Palaearctic | yes | diverse | long-distance | invertebrates | high | landbirds | genus |
| Apodiformes | Trochilidae | Calypte | Nearctic | no | diverse | partial | nectarivore | medium | landbirds | genus |
| Columbiformes | Columbidae | Columba | Holarctic | yes | diverse | resident | omnivore | high | landbirds | genus |
| Columbiformes | Columbidae | Streptopelia | Palaearctic | yes | open | long-distance | herbivore | high | landbirds | genus |
| Coraciiformes | Cerylidae | Chloroceryle | Neotropic | no | forest | resident | piscivore | low | landbirds | family |
| Coraciiformes | Meropidae | Merops | Palaearctic | no | open | full migrant | invertebrates | low | landbirds | genus |
| Cuculiformes | Cuculidae | Ceuthmochares | African | no | forest | resident | invertebrates | low | landbirds | family |
| Cuculiformes | Cuculidae | Cuculus | Palaearctic | yes | diverse | long-distance | invertebrates | high | landbirds | genus |
| Passeriformes | Hirundinidae | Progne | Palaearctic | no | diverse | partial | invertebrates | medium | landbirds | genus |
| Passeriformes | Corvidae | Corvus | Holarctic | yes | diverse | resident | omnivore | low | landbirds | genus |
| Passeriformes | Alaudidae | Eremophila | Holarctic | yes | open | long-distance | omnivore | high | landbirds | genus |
| Passeriformes | Fringillidae | Leucosticte | Palaearctic | yes | open | resident | herbivore | high | landbirds | genus |
| Passeriformes | Fringillidae | Serinus | Palaearctic | no | diverse | resident | herbivore | medium | landbirds | genus |
| Passeriformes | Hirundinidae | Cecropis | Palaearctic | yes | diverse | long-distance | invertebrates | high | landbirds | genus |
| Passeriformes | Hirundinidae | Hirundo | Holarctic | yes | open | long-distance | invertebrates | high | landbirds | genus |
| Passeriformes | Icteridae | Agelaius | Nearctic | yes | water | partial | omnivore | high | landbirds | genus |
| Passeriformes | Icteridae | Molothrus | Nearctic | no | open | medium-distance | omnivore | medium | landbirds | genus |
| Passeriformes | Motacillidae | Motacilla | Palaearctic | yes | open | partial | invertebrates | high | landbirds | genus |
| Passeriformes | Muscicapidae | Ficedula | Palaearctic | yes | forest | long-distance | invertebrates | medium | landbirds | genus |
| Passeriformes | Muscicapidae | Oenanthe | Palaearctic | yes | open | long-distance | invertebrates | medium | landbirds | genus |
| Passeriformes | Paridae | Cyanistes | Palaearctic | yes | diverse | resident | invertebrates | medium | landbirds | genus |
| Passeriformes | Paridae | Parus | Palaearctic | yes | forest | resident | invertebrates | medium | landbirds | genus |
| Passeriformes | Paridae | Pseudopodoces | Palaearctic | no | open | resident | herbivore | medium | landbirds | genus |
| Passeriformes | Passerellidae | Melospiza | Nearctic | no | water | medium-distance | invertebrates | low | landbirds | genus |
| Passeriformes | Passerellidae | Melozone | Nearctic | no | shrubland | resident | herbivore | low | landbirds | family |
| Passeriformes | Passerellidae | Zonotrichia | Nearctic | yes | shrubland | medium-distance | herbivore | medium | landbirds | genus |
| Passeriformes | Passeridae | Onychostruthus | Palaearctic | no | open | resident | herbivore | high | landbirds | genus |
| Passeriformes | Passeridae | Passer | Palaearctic | yes | diverse | resident | herbivore | medium | landbirds | genus |
| Passeriformes | Passeridae | Pyrgilauda | Palaearctic | no | open | resident | herbivore | high | landbirds | genus |
| Passeriformes | Sturnidae | Sturnus | Palaearctic | yes | diverse | partial | invertebrates | high | landbirds | genus |
| Passeriformes | Turdidae | Catharus | Nearctic | no | forest | long-distance | omnivore | medium | landbirds | genus |
| Passeriformes | Turdidae | Erithacus | Palaearctic | yes | forest | partial | invertebrates | high | landbirds | genus |
| Passeriformes | Tyrannidae | Empidonax | Nearctic | no | forest | long-distance | invertebrates | medium | landbirds | genus |
| Passeriformes | Tyrannidae | Myiozetetes | Neotropic | no | forest | resident | invertebrates | low | landbirds | family |
| Piciformes | Picidae | Dryobates | Nearctic | yes | forest | resident | invertebrates | medium | landbirds | genus |
| Ciconiiformes | Pteroclidae | Pterocles | African | no | open | resident | herbivore | medium | sandgrouses | family |
| Galliformes | Phasianidae | Centrocercus | Nearctic | yes | open | resident | herbivore | low | chicken birds | genus |
| Galliformes | Phasianidae | Meleagris | Nearctic | yes | diverse | resident | herbivore | low | chicken birds | order |
| Galliformes | Phasianidae | Coturnix | Palaearctic | yes | open | medium-distance | herbivore | high | chicken birds | genus |
| Galliformes | Phasianidae | Crossoptilon | Palaearctic | yes | forest | resident | herbivore | medium | chicken birds | genus |
| Galliformes | Phasianidae | Gallus | Holarctic | yes | forest | resident | herbivore | medium | chicken birds | order |
| Galliformes | Phasianidae | Lagopus | Holarctic | yes^3,4^ | open | resident | herbivore | high | chicken birds | genus |
| Galliformes | Phasianidae | Phasianus | Holarctic | yes | open | resident | herbivore | high | chicken birds | genus |
| Galliformes | Phasianidae | Tetrastes | Palaearctic | yes | forest | resident | herbivore | high | chicken birds | genus |
| Galliformes | Phasianidae | Tympanuchus | Nearctic | yes^3,4^ | open | resident | herbivore | medium | chicken birds | genus |

**Table S1.2 Plant taxon selection list** (Streptophyta).

| Family | Growth form | Growth form simplified | Family | Growth form | Growth form simplified |
| --- | --- | --- | --- | --- | --- |
| Haloragaceae | aquatic | aquatic | **Crassulaceae** | herb | forbs_and_grasses |
| Hydrocharitaceae | aquatic | aquatic | **Cyperaceae** | grass | forbs_and_grasses |
| Potamogetonaceae | aquatic | aquatic | **Droseraceae** | herb | forbs_and_grasses |
| Diapensiaceae | cushion_shrub | cushion_shrub | **Equisetaceae** | herb | forbs_and_grasses |
| Acoraceae | herb | forbs_and_grasses | **Euphorbiaceae** | herb | forbs_and_grasses |
| Adoxaceae | herb | forbs_and_grasses | **Fabaceae** | herb | forbs_and_grasses |
| Amaranthaceae | herb | forbs_and_grasses | **Gentianaceae** | herb | forbs_and_grasses |
| Amaryllidaceae | herb | forbs_and_grasses | **Geraniaceae** | herb | forbs_and_grasses |
| Apiaceae | herb | forbs_and_grasses | **Hyacinthaceae** | herb | forbs_and_grasses |
| Araliaceae | herb | forbs_and_grasses | **Hydrophyllaceae** | herb | forbs_and_grasses |
| Aristolochiaceae | herb | forbs_and_grasses | **Iridaceae** | herb | forbs_and_grasses |
| Asphodelaceae | herb | forbs_and_grasses | **Juncaceae** | grass | forbs_and_grasses |
| Asteraceae | herb | forbs_and_grasses | **Juncaginaceae** | grass | forbs_and_grasses |
| Balsaminaceae | herb | forbs_and_grasses | **Lentibulariaceae** | herb | forbs_and_grasses |
| Boraginaceae | herb | forbs_and_grasses | **Liliaceae** | herb | forbs_and_grasses |
| Brassicaceae | herb | forbs_and_grasses | **Linaceae** | herb | forbs_and_grasses |
| Campanulaceae | herb | forbs_and_grasses | **Malvaceae** | herb | forbs_and_grasses |
| Caprifoliaceae | herb | forbs_and_grasses | **Melanthiaceae** | herb | forbs_and_grasses |
| Caryophyllaceae | herb | forbs_and_grasses | **Montiaceae** | herb | forbs_and_grasses |
| Celastraceae | herb | forbs_and_grasses | **Nartheciaceae** | herb | forbs_and_grasses |
| Comandraceae | herb | forbs_and_grasses | **Onagraceae** | herb | forbs_and_grasses |
| Convolvulaceae | herb | forbs_and_grasses | **Orchidaceae** | herb | forbs_and_grasses |
| Cornaceae | herb | forbs_and_grasses | **Orobanchaceae** | herb | forbs_and_grasses |
| Oxalidaceae | herb | forbs_and_grasses | **Apocynaceae** | woody | woody |
| Paeoniaceae | herb | forbs_and_grasses | **Aquifoliaceae** | tree | tree |
| Papaveraceae | herb | forbs_and_grasses | **Cupressaceae** | tree | tree |
| Phrymaceae | herb | forbs_and_grasses | **Oleaceae** | tree | tree |
| Plantaginaceae | herb | forbs_and_grasses | **Pinaceae** | tree | tree |
| Family | Growth form | Growth form simplified | Family | Growth form | Growth form simplified |
| Plumbaginaceae | herb | forbs_and_grasses | **Sapindaceae** | tree | tree |
| Poaceae | grass | forbs_and_grasses | **Ulmaceae** | tree | tree |
| Polemoniaceae | herb | forbs_and_grasses | **Berberidaceae** | woody | woody |
| Polygalaceae | herb | forbs_and_grasses | **Betulaceae** | woody | woody |
| Polygonaceae | herb | forbs_and_grasses | **Elaeagnaceae** | woody | woody |
| Portulacaceae | herb | forbs_and_grasses | **Ephedraceae** | woody | woody |
| Primulaceae | herb | forbs_and_grasses | **Ericaceae** | woody | woody |
| Ranunculaceae | herb | forbs_and_grasses | **Grossulariaceae** | woody | woody |
| Resedaceae | herb | forbs_and_grasses | **Hypericaceae** | woody | woody |
| Rosaceae | herb | forbs_and_grasses | **Lamiaceae** | woody | woody |
| Rubiaceae | herb | forbs_and_grasses | **Lythraceae** | woody | woody |
| Sarraceniaceae | herb | forbs_and_grasses | **Myricaceae** | woody | woody |
| Saxifragaceae | herb | forbs_and_grasses | **Rhamnaceae** | woody | woody |
| Scrophulariaceae | herb | forbs_and_grasses | **Salicaceae** | woody | woody |
| Solanaceae | herb | forbs_and_grasses | **Tamaricaceae** | woody | woody |
| Thymelaeaceae | herb | forbs_and_grasses | **Taxaceae** | woody | woody |
| Tofieldiaceae | herb | forbs_and_grasses |  |  |  |
| Urticaceae | herb | forbs_and_grasses |  |  |  |
| Violaceae | herb | forbs_and_grasses |  |  |  |
| Vitaceae | herb | forbs_and_grasses |  |  |  |

**Table S2.1** List of the 49 genera included in PCA and dbRDA analyses.

| **Genus** | **Taxon reads** | **%** | **Genus** | **Taxon reads** | **%** |
| --- | --- | --- | --- | --- | --- |
| Phasianus | 862 | 6,11 | Columba | 218 | 1,54 |
| Lagopus | 678 | 4,80 | Myiozetetes | 216 | 1,53 |
| Falco | 520 | 3,68 | Onychostruthus | 214 | 1,52 |
| Corvus | 508 | 3,60 | Zonotrichia | 213 | 1,51 |
| Oenanthe | 474 | 3,36 | Apus | 202 | 1,43 |
| Calypte | 460 | 3,26 | Rissa | 201 | 1,42 |
| Cyanistes | 454 | 3,22 | Antrostomus | 197 | 1,40 |
| Catharus | 443 | 3,14 | Fulmarus | 194 | 1,37 |
| Parus | 426 | 3,02 | Pyrgilauda | 192 | 1,36 |
| Dryobates | 423 | 3,00 | Grus | 185 | 1,31 |
| Serinus | 405 | 2,87 | Oxyura | 183 | 1,30 |
| Ficedula | 401 | 2,84 | Tympanuchus | 162 | 1,15 |
| Hirundo | 399 | 2,83 | Gymnogyps | 138 | 0,98 |
| Pseudopodoces | 347 | 2,46 | Empidonax | 131 | 0,93 |
| Cuculus | 336 | 2,38 | Calidris | 120 | 0,85 |
| Agelaius | 320 | 2,27 | Chaetura | 120 | 0,85 |
| Molothrus | 291 | 2,06 | Merops | 117 | 0,83 |
| Anser | 288 | 2,04 | Accipiter | 112 | 0,79 |
| Cygnus | 285 | 2,02 | Aquila | 109 | 0,77 |
| Sturnus | 281 | 1,99 | Nipponia | 109 | 0,77 |
| Anas | 280 | 1,98 | Pelecanus | 102 | 0,72 |
| Aythya | 277 | 1,96 |  |  |  |
| Melozone | 271 | 1,92 |  |  |  |
| Motacilla | 271 | 1,92 |  |  |  |
| Melospiza | 267 | 1,89 |  |  |  |
| Tyto | 247 | 1,75 |  |  |  |
| Coturnix | 246 | 1,74 |  |  |  |
| Passer | 219 | 1,55 |  |  |  |

**Table S2.2** Importance of components in PCA for the relative abundances of 49 bird genera.

| Component | PC1 | PC2 | PC3 | PC4 | PC5 |
| --- | --- | --- | --- | --- | --- |
| Standard deviation | 0.19 | 0.15 | 0.14 | 0.13 | 0.12 |
| Proportion of Variance | 0.10 | 0.06 | 0.05 | 0.04 | 0.04 |
| Cumulative Proportion | 0.10 | 0.16 | 0.21 | 0.25 | 0.29 |

**Table S3** dbRDA evaluation results for three different models for the hellinger-transformed taxon reads of 49 bird genera (response). Partitioning of squared Euclidean distance and p-values from permutation tests (function anova).

| Model (explanatory variables) | Total | Proportion unconstrained | Proportion constrained | p-value |
| --- | --- | --- | --- | --- |
| ~ years BP + lake + forbs_and_grasses + trees + aquatic | 38.0 | 0.83 | 0.17 | <0.01 |
| ~ years BP + lat + forbs_and_grasses + trees + aquatic | 38.0 | 0.92 | 0.08 | <0.01 |
| ~ lake | 38.0 | 0.89 | 0.11 | <0.01 |
| ~ years BP | 38.0 | 0.98 | 0.02 | <0.01 |

**
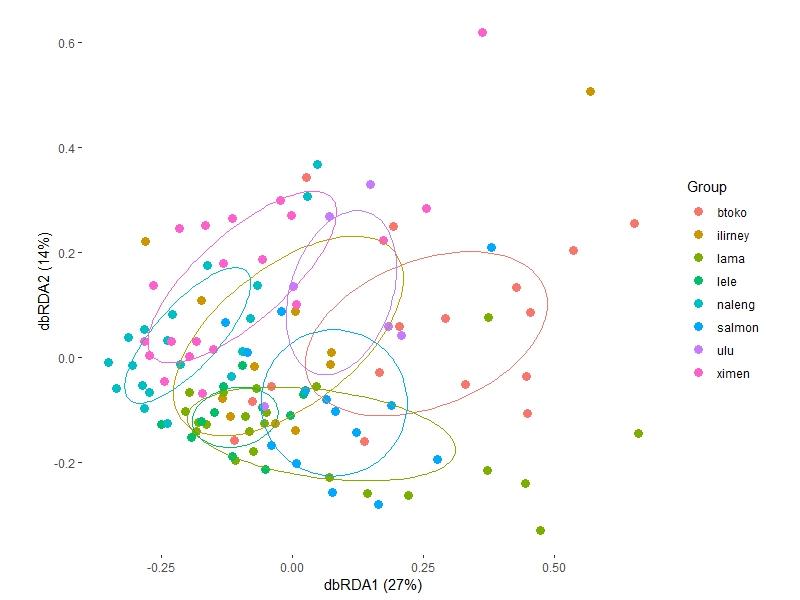

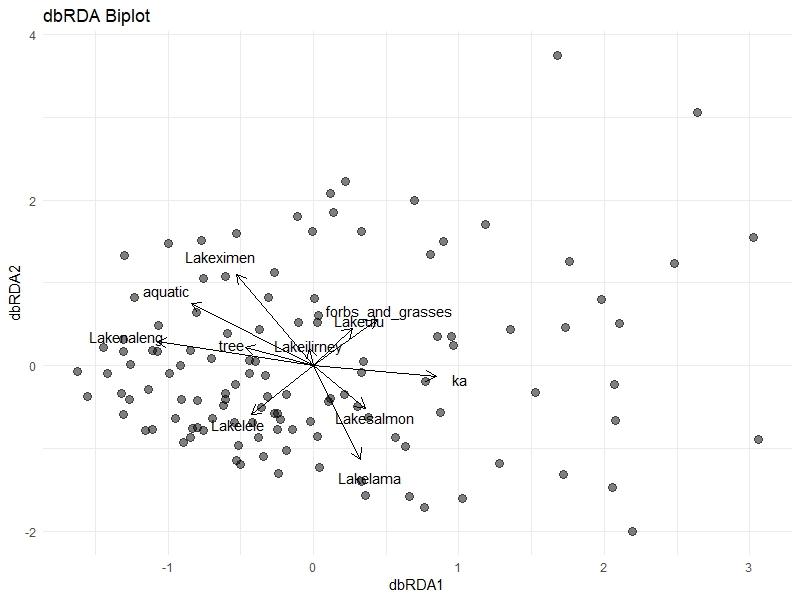
**

**Figure S2 A** Ordiplot (function *gg_ordiplot* with kind = “sd”, package ggordiplots) of the dbRDA shows a clustering of bird taxa occurrence according to the sampling location (lakes). Large overlaps are visible for the lakes at the southern edge of the Palaearctic, Lakes Ximen and Naleng, as well as for the easternmost Palaearctic and the westernmost Nearctic lakes Ilirney and Salmon. The northernmost lake Levinson-Lessing shows the smallest variation in bird taxa abundance, while Lama and Bolshoe-Toko show higher variation (size of group ellipses = standard deviation). **B** Drivers of bird community identified by the dbRDA. Details are given in Table 3, Table S3.

**
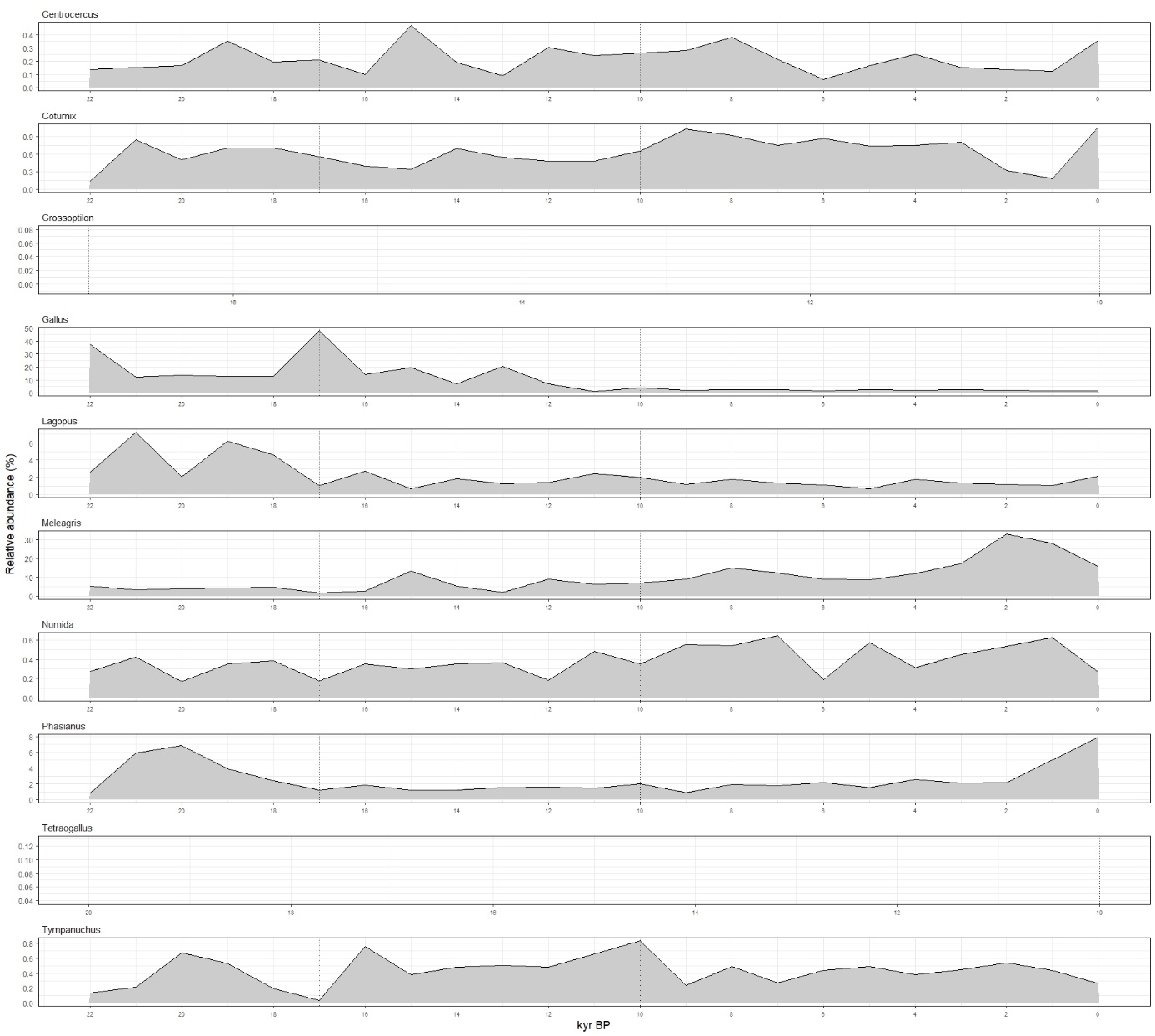
**

**Figure S3** Major shifts in relative sedaDNA read assignment by shotgun sequencing through time for eight northern high-latitude and high-elevation lake cores. Chicken birds (Galliformes) split into the genera present in the dataset. Relative abundance: taxon reads per genus and year as % of all Aves taxon reads. Note significant differences in rel. abundances (y-axis).

**
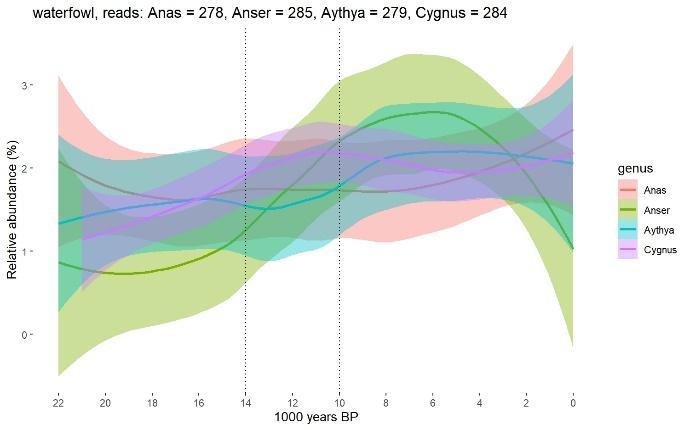
**
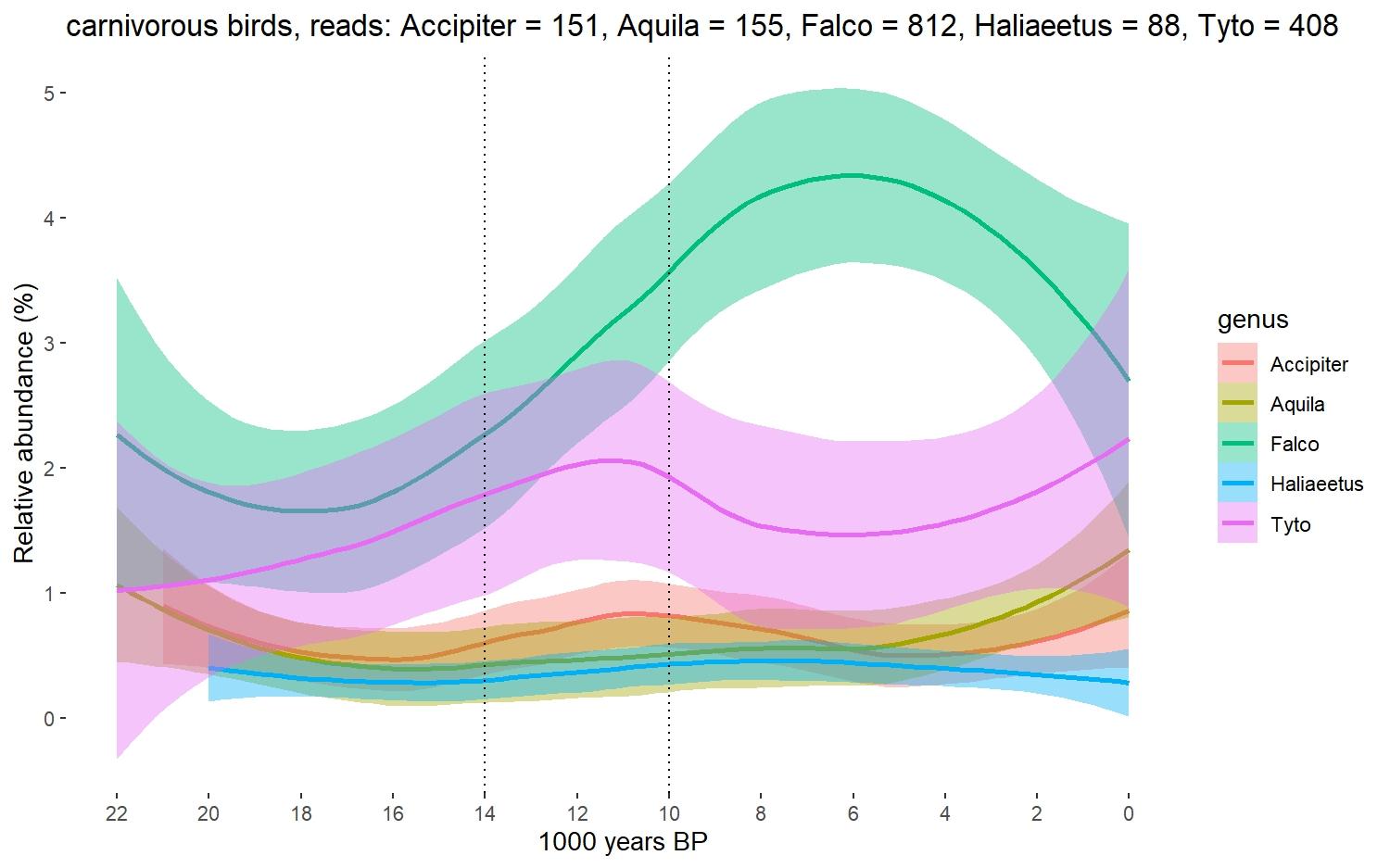


**Figure S4** Abundance shifts in waterfowl (left) and raptors and owls (right) split into the genera present in the dataset, across time for all eight arctic lakes plotted with ggplot function *geom_smooth* (method = “loess”).


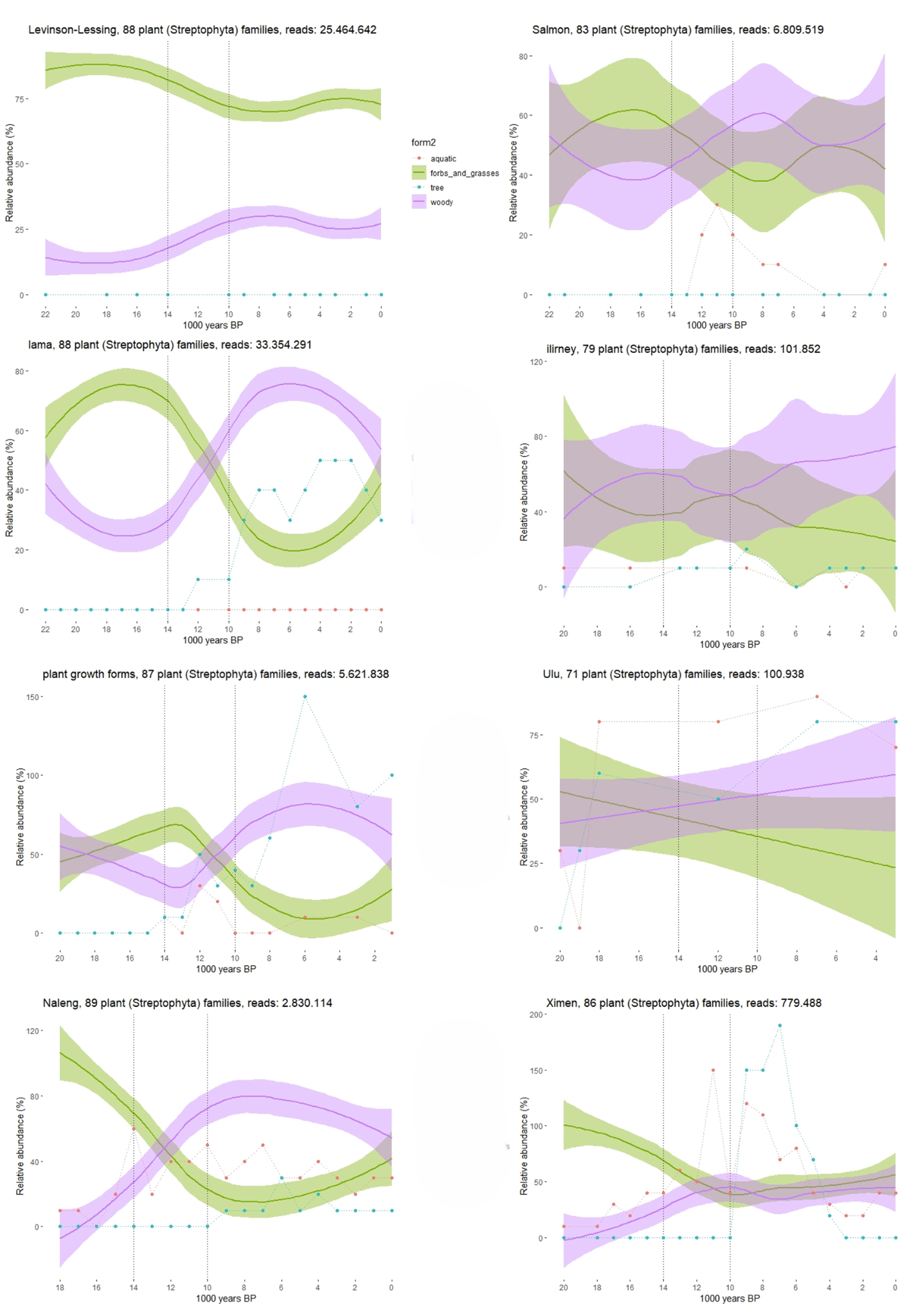


**Figure S5** Abundance shifts in plant growth forms across time for each lake sediment core, plotted with ggplot function *geom_smooth* (method = “loess”).

**
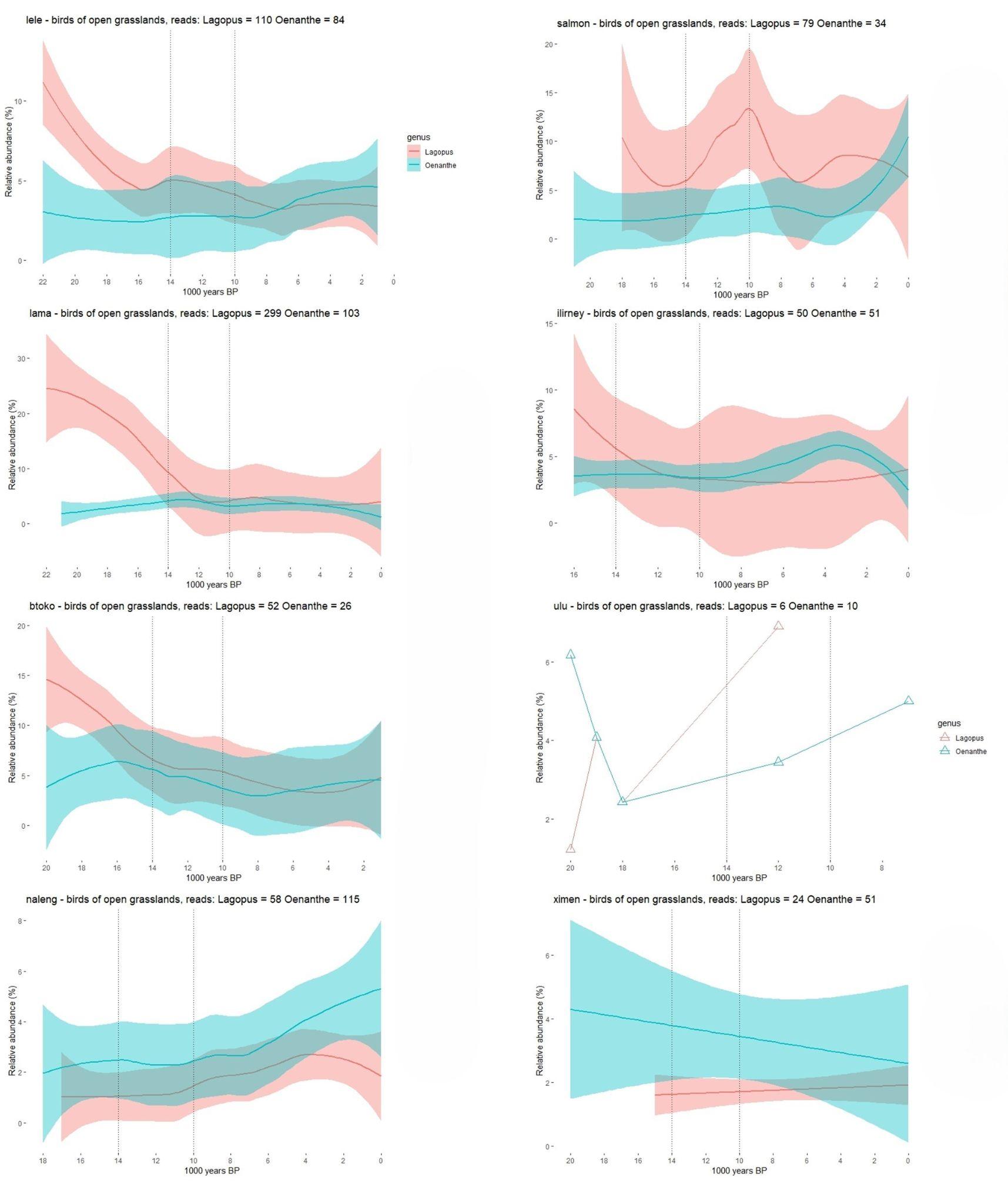
**

**Figure S6** Abundance shifts in representatives of open grasslands across time for each lake sediment core, plotted with ggplot function *geom_smooth* (method = “loess”), except for lake Ulu (no model) and Ximen (method = “glm”) due to low read counts.

**
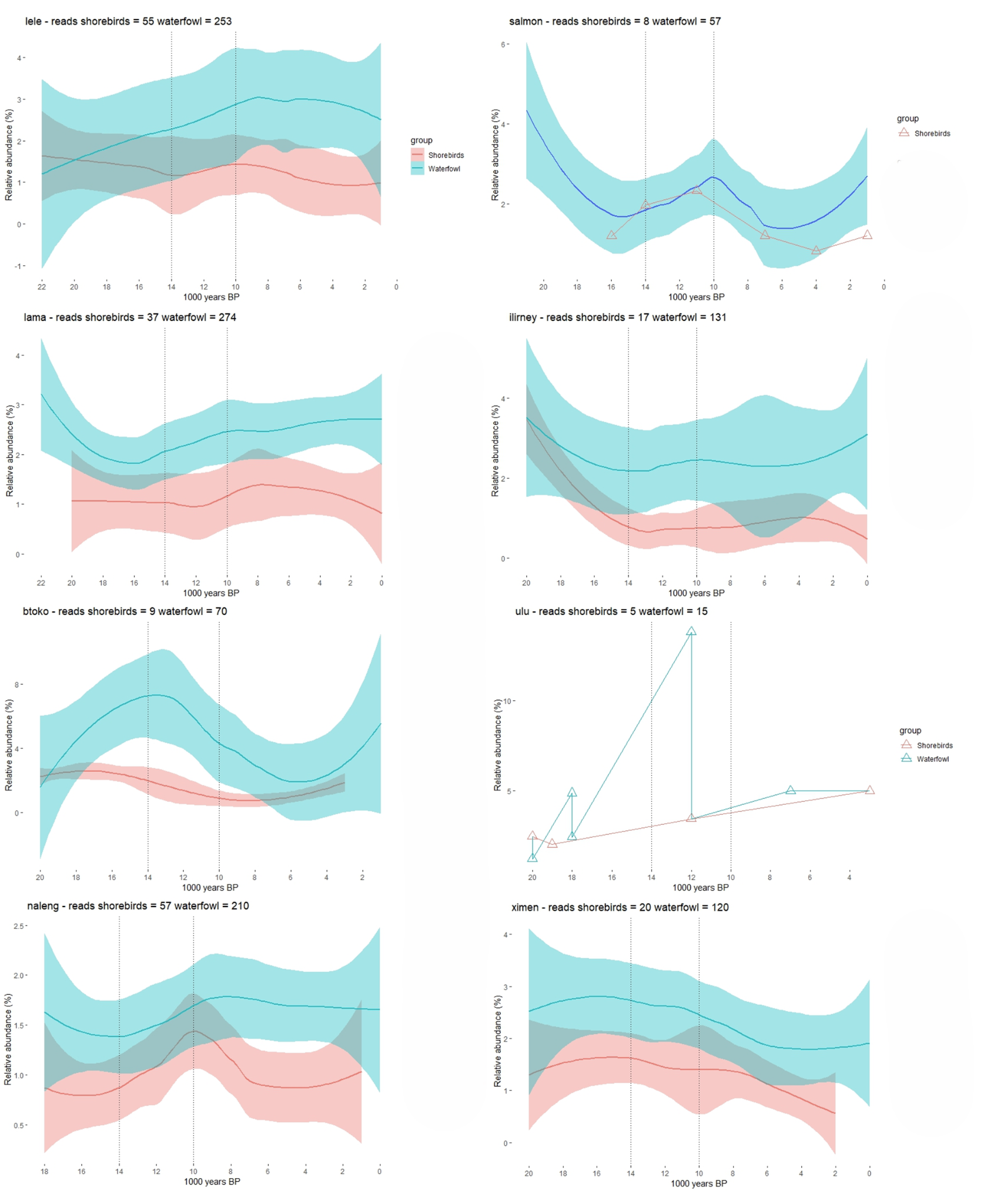
**

**Figure S7** Abundance shifts in shorebirds (*Charadrius*, *Calidris*) and waterfowl (Anseriformes) across time for each lake sediment core, plotted with ggplot function *geom_smooth* (method = “loess”), except for lake Ulu (no model) and Lake Salmon (for shorebirds) due to low read counts.
